# Nanoelectronic Detection of Opioids: Machine Learning‐Powered Screening With Carbon Nanotube Field‐Effect Transistor Sensor Array

**DOI:** 10.1002/smll.74244

**Published:** 2026-06-19

**Authors:** Zhengru Liu, Mehdy Dousty, Wenting Shao, Jieyu Wang, Samia Afrin, Wuqi Li, Ervin Sejdić, Alexander Star

**Affiliations:** ^1^ Department of Chemistry University of Pittsburgh Pittsburgh Pennsylvania USA; ^2^ The Edward S. Rogers Department of Electrical and Computer Engineering University of Toronto Toronto Ontario Canada; ^3^ North York General Hospital Toronto Ontario Canada; ^4^ Department of Bioengineering University of Pittsburgh Pittsburgh Pennsylvania USA

**Keywords:** carbon nanotube, field‐effect transistor, laboratory automation, machine learning, opioid sensing

## Abstract

The development of inexpensive, portable, and rapid opioid screening techniques is urgently needed because of the ongoing opioid crisis in the United States, primarily driven by illicit fentanyl. In this work, a nanoelectronic detection method was developed for opioids using machine learning. Fentanyl and three other opioids (codeine, hydrocodone, and morphine) were tested with field‐effect transistor (FET) sensor array composed of single‐walled carbon nanotubes (SWCNTs) decorated with gold and platinum nanoparticles. Hundreds of opioid samples were tested manually and with an automated electrolyte‐gated FET test system capable of testing up to 96 sensors. Fifteen features were extracted from experimental FET data to train conventional supervised machine learning models. Optimized linear discriminant analysis (LDA) and random forest (RF) models were applied for opioid classification, achieving 82.6% accuracy. Deep learning methods were subsequently applied to the full FET dataset to eliminate human bias in feature selection. A convolutional neural network (CNN) achieved 98.3% accuracy for the manual test group and revealed the significant role of gate‐source current in distinguishing opioid molecules. This insight guided improvements to conventional models, uncovering unexpected mechanisms of molecular sensing with liquid‐gated FETs and demonstrating the broader potential of combining nanoelectronics with machine learning for chemical detection.

## Graphical abstract

A carbon nanotube field‐effect transistor (NTFET) sensor array is constructed to screen different opioids with machine learning models. Codeine, fentanyl, hydrocodone, and morphine are tested using the NTFET sensor array and classified with both conventional supervised learning models and deep learning models. Deep learning models reveal the significant role of gate‐source current in opioid discrimination.

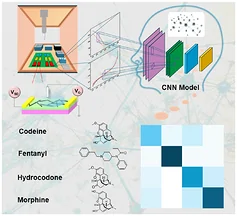

## 1 Introduction

Some natural opioids (e.g., morphine and codeine) and synthetic opioids (e.g., fentanyl and methadone) are widely used as pain relievers in medical treatment. However, the euphoria induced by opioids could lead to misuse and overdose, which is often lethal because of respiratory depression. In recent years, opioid abuse has become a public health concern, culminating in the declaration of an opioid crisis in the United States in 2017. This public health emergency has been primarily driven by the illegal dealings and use of opioids such as heroin, morphine, and especially a more potent synthetic opioid, fentanyl. As a result, in addition to stricter restrictions on opioids prescriptions, effective monitoring and tracking of opioids are essential to limit illicit manufacturing and distribution. This need has created strong demand for high‐throughput analytical methods capable of addressing the rapidly increasing volume of opioid testing required during the ongoing opioid crisis.

The determination of opioids can be realized with different instrumental analytical techniques, including optical methods [1, 2, 3], mass spectrometry [4, 5, 6], and electrochemical methods [7, 8, 9]. Among these, chromatographic methods coupled with mass spectrometry currently are the most powerful strategies for quantitative analysis of opioids [10, 11, 12, 13, 14]. These techniques enable efficient separation of opioids from complex matrices and precise identification with high‐resolution mass spectrometry. However, hyphenated chromatographic methods are usually time‐consuming, require skilled personnel, and are largely limited to laboratory settings because of bulky instrumentation and high operational costs. The optical methods have high sensitivity and good capacity for real‐time monitoring in vivo [15], while an extra labeling procedure may be still needed for the recognition of opioids. For routine monitoring applications, the screening of opioids during manufacturing and distribution, such as the rapid identification of opioids in powder mixtures and oral or injectable solutions, is also of great importance for preventing opioid overdose. It is therefore essential to develop methods for the rapid screening of opioids even with a compromise of sensitivity. Electrochemical methods offer rapid screening with high sensitivity and have been widely explored for opioid detection using techniques such as cyclic voltammetry [16, 17, 18], square‐wave voltammetry [19, 20, 21], differential pulse voltammetry [18, 22], and field‐effect transistors (FETs) [23, 24, 25, 26]. Nevertheless, achieving sufficient selectivity remains a major challenge in electrochemical opioid sensing, as false‐positive responses can arise from interfering species. Although specificity could be improved with the use of capture probes such as antibodies [24] and aptamers [21, 23, 27], discriminating among structurally similar opioids remains difficult because they often share common binding sites.

Machine learning methods offer new opportunities for distinguishing opioids with similar chemical structures. In recent years, machine learning has been increasingly utilized for opioid classification by analyzing “sample fingerprints”, which consist of multiple characteristic features extracted from raw data [28, 29, 30, 31, 32]. In contrast to traditional methods that rely on a limited number of predefined parameters, these methods use a broader set of features to enhance discrimination. Although most reported studies employ spectrometric techniques that are not portable, labor‐intensive, and time‐consuming, they clearly demonstrate the great potential of machine learning methods in opioid screening. Importantly, model performance depends heavily on the availability of sufficient training data, necessitating a large number of measurements for each compound.

FET‐based sensors are good choices to achieve this goal. The miniaturized size and high level of integration make FET sensors especially attractive as sensor units for high‐throughput opioid analysis. Interactions between opioid molecules and the sensing layers induce changes in transistor electrical parameters, generating large datasets that can be used to construct unique fingerprints for each sample and further analyzed with different machine learning algorithms. In our previous work, we reported carbon nanotube field‐effect transistor (NTFET) sensor arrays for the classification of different target analytes, including purine compounds [33] and cells [34], using 15 features extracted from the FET transfer characteristics. However, a single transfer characteristic curve can contain hundreds of data points, and substantial information is lost during feature extraction. Moreover, models established based on a limited number of manually selected features are intrinsically prejudiced, since these features are selected based on previously proposed mechanisms of analyte detection with carbon nanotube (CNT) transistors [35]. Therefore, there is a clear need to develop new models that incorporate richer datasets while minimizing prior assumptions.

In this work, we report the construction of a NTFET sensor array for screening four opioids with machine learning methods. The FET sensors were fabricated with semiconducting single‐walled carbon nanotubes (SWCNTs) decorated with metal nanoparticles and applied for the detection of codeine, fentanyl, hydrocodone, and morphine. The use of non‐specific sensors eliminates the need for antibodies or aptamers at the sensing interface, enhancing sensor robustness and extending shelf life. Combined with machine learning, this approach enables discrimination among different opioids. We investigated opioid screening with different testing approaches and evaluated different classification models. In addition to manual testing, an automated electrolyte‐gated FET platform was employed to enable high‐throughput testing of the sensor array. Initial classification was performed with conventional supervised machine learning models based on 15 features extracted from the source‐drain current (I_ds_) with an accuracy value of 82.6%. Subsequently, deep learning methods were introduced for the classification of opioids by utilizing the full FET dataset. Using an optimized multi‐path convolutional neural network (CNN) method, the classification accuracy for four opioids reached 98.3% in the manual test group. More importantly, the CNN model revealed critical importance of gate‐source current (I_gs_) in the classification of opioids, providing guidance for improving the conventional supervised learning models by adding I_gs_ related features and realizing higher accuracy (89.0%). These findings highlight the significance of I_gs_ to improve the classification and offer valuable insights for the design of future NTFET‐based sensor arrays.

## 2 Experimental Section

The NTFET sensors were fabricated with commercial semiconductor‐enriched SWCNTs (IsoSol‐S100, NanoIntegris) with 0.1% metallic and 99.9% semiconducting composition. The metal nanoparticles were decorated on the bare CNT by bulk electrolysis. 1 mm chloroauric acid (HAuCl_4_·3H_2_O, Alfa Aesar) and 1 mm chloroplatinic acid (HPtCl_6_·xH_2_O, Sigma–Aldrich) were prepared in 0.1 m HCl solution for bulk electrolysis. The certified reference standards (1 mg mL^−1^ in methanol) of codeine, fentanyl, hydrocodone, and morphine were all purchased from Cerilliant.

### 2.1 Fabrication of NTFET

The standard 40‐pin ceramic dual inline packages were used for wire bonding with 2.6 × 2.6 mm^2^ chips, which were fabricated with Si/SiO_2_ wafers patterned with interdigitated gold electrodes (39 channels with a channel length of 6 µm, shown in Figure S1) using photolithography. The wire‐bonded packages were secured with polydimethylsiloxane (PDMS) and heated at 200°C for 1 h. The IsoSol‐SWCNTs (3 µL, 0.02 g mL^−1^, dispersed in toluene) were then deposited between the interdigitated electrodes through dielectrophoresis (DEP) using a Keithley 3390 Arbitrary Waveform (10 V_PP_, 100 kHz for 2 min). The chips deposited with SWCNTs were then annealed at 200°C for 24 h on a hotplate to improve the contact between the SWCNTs and the gold electrodes. The bulk electrolysis of HAuCl_4_·3H_2_O/ HPtCl_6_·*x*H_2_O was realized by an electrochemical analyzer (CH instruments). A 1 m Ag/AgCl electrode was used as the reference electrode in the bulk electrolysis, Pt electrode worked as the counter electrode, and SWCNTs on the chip were applied as the working electrode.

### 2.2 Characterization

XplorA Raman‐AFM/TERS system was employed for the collection of Raman spectra using a 638 nm (24 mW) laser for excitation, which was operated at 1% power. The Raman Spectra were collected from 25 different locations on a sensor chip and averaged for plotting. All spectra were normalized to the Si peak at 499.8 nm for the comparison of spectra among different samples. A Zeiss Sigma 500 VP Scanning Electron Microscope was employed for the characterization of the surface morphology of the CNT hybrids. The atomic force microscopy images were taken by a Bruker Multimode eight AFM system with a Veeco Nanoscope III a controller in the tapping mode. The AFM images were processed by Gwyddion.

### 2.3 Opioid Sensing With Manual Operations

The NTFET sensors were immersed in 400 µL of 0.01 m phosphate buffer saline (PBS) for 10 min, and the above step was repeated three times. After rinsing with nanopure water and drying, the sensors were incubated in another 400 µL of PBS for 1 h and the NTFET characteristics were measured as the control group. A Keithley Source Meter Unit 2400 was applied for electric measurements. To avoid opioid oxidation and water splitting at positive voltages [18, 36], the gate voltage was swept from 0.6 to −0.6 V with an interval of 0.06 V, and the source‐drain voltage is 0.05 V. Both I_ds_ values and I_gs_ values at 200 different voltage values were recorded. For each measurement, the sensor was rinsed with nanopure water first and dried with nitrogen gas flow, then immersed in 400 µL of PBS for 2 min before the measurement started. Then, the same sensor was incubated with 1 µg of opioid reference standard (10 µg mL^−1^, 100 µL in PBS) for 1 h. After rinsing and drying, the NTFET characteristics were measured as the experimental group. After tests, the sensors were recovered by rinsing with isopropanol and stored for the test of another compound. The data collected manually was assigned as the manual test group. For each compound, all the samples are tested with separate sensors (e.g., 43 codeine samples are tested with 43 different sensors), and the test protocol is summarized in Figure S2.

### 2.4 Opioid Sensing With an Automated Electrolyte‐Gated FET Test System

**FIGURE 1:**
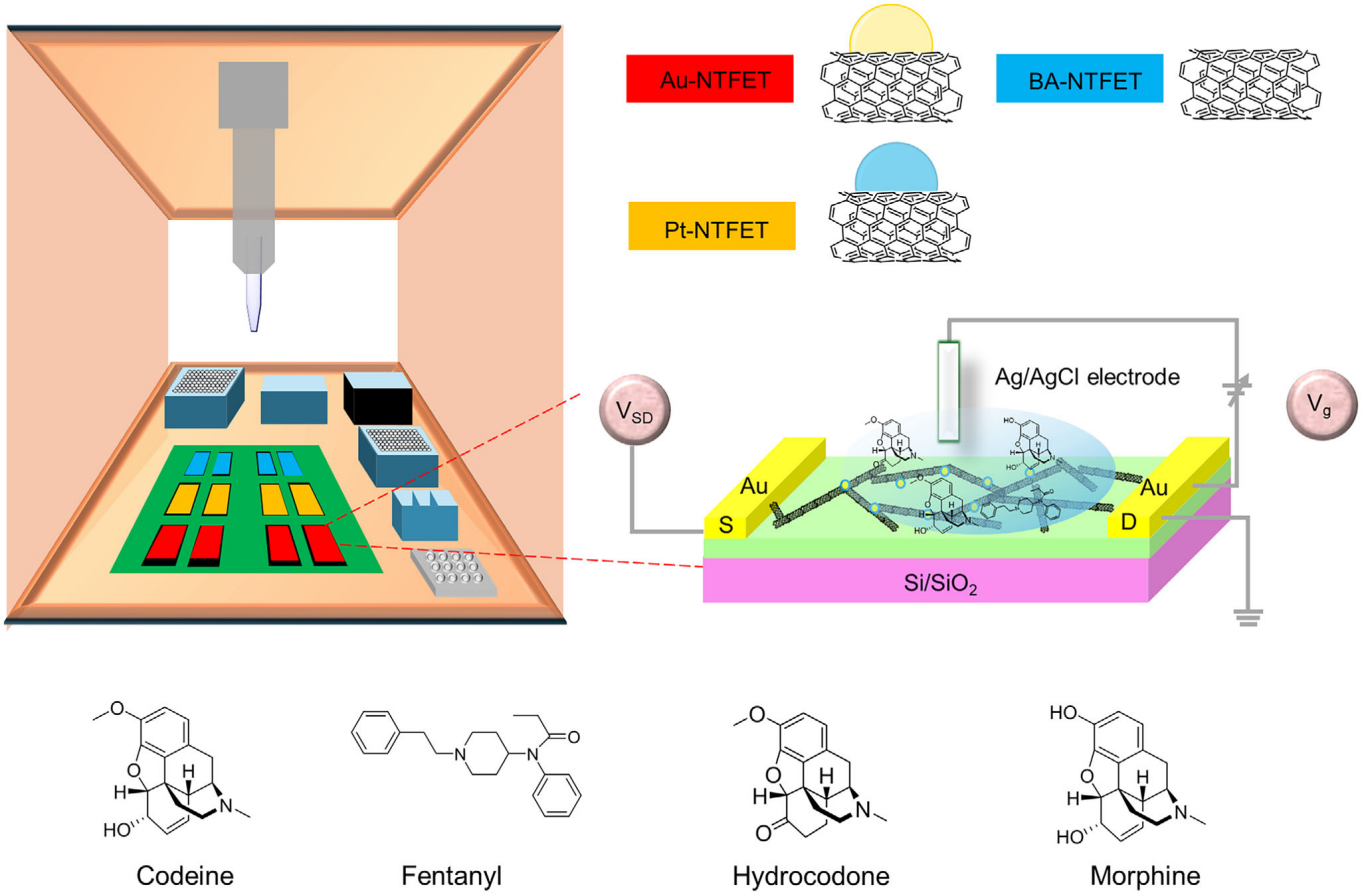
Schematic illustration of opioid screening with the automated electrolyte‐gated FET test system. The left panel shows the automated system, and the right panel depicts an electrolyte‐gated FET. The sensor array consisted of three types of NTFET sensors: bare SWCNT devices (BA‐NTFET) and SWCNT devices decorated with gold nanoparticles (Au‐NTFET) or platinum nanoparticles (Pt‐NTFET).

The opioid sensing using the automated electrolyte‐gated FET test system shares a similar protocol with the manual test group but with several changes, which are clarified in Figure S3, and the Python script to run the test is listed in Appendix B. The details of the automated test system are discussed in another publication [37]. In each experiment, a calibration protocol was executed before running the automated system, including calibration of the pipette, electrode position, and deck position. The sensors were incubated with 240 µL of PBS, which was transferred by the pipetting robot, for 10 mins and repeated 3 times. Then these sensors were incubated with 240 µL of PBS for 1 h. Since gas drying hadn't been coupled with the automated system, a solution removal protocol was developed to remove the residual on the sensor thoroughly before the electrical measurement. The pipette tip aspirated solution on 25 different sections on the package to remove all the residuals. The NTFET characteristics as the control group were then measured in 240 µL of PBS after incubation for 2 mins. A Keithley Source Meter Unit 2600 was applied as the detector in the automated system. Subsequently, the pipetting robot transferred 1 µg of opioid reference standard (10 µg mL^−1^, 100 µL in PBS) to the sensor. After 1 h incubation, the sensors were rinsed with 240 µL of PBS twice and residual was removed thoroughly. Finally, the NTFET characteristics as the experimental group were measured. The sensors were also recovered by rinsing with isopropanol. The data collected with the automated system was assigned as the automated test group. Scheme for the automated system is presented in Figure 1.

### 2.5 Classification of Different Opioids With Conventional Supervised Learning Algorithms

For the quality control, the transfer characteristics of sensors used for data analysis should fulfill all three requirements: a) I_ds @ 0.6 V_ <10^−5^ A; b) I_ds @ −0.6 V_ > 10^−5^ A; c) I_ds @ −0.6 V_/I_ds @ 0.6 V_ >10. All the data analysis in this work was done with Python, and the codes are listed in Appendix A. First, features listed in Table S1 were extracted from the NTFET characteristics of the control group and the experimental group. There are 43 samples for each compound in the manual test group and 41 samples in the automated test group. For the classification of different opioids, two different algorithms, linear discriminant analysis (LDA) and random forest (RF) were applied for model training. The leave‐one‐out method was used for the cross validations.

### 2.6 Classification of Different Opioids With Deep Learning Methods

**FIGURE 2:**
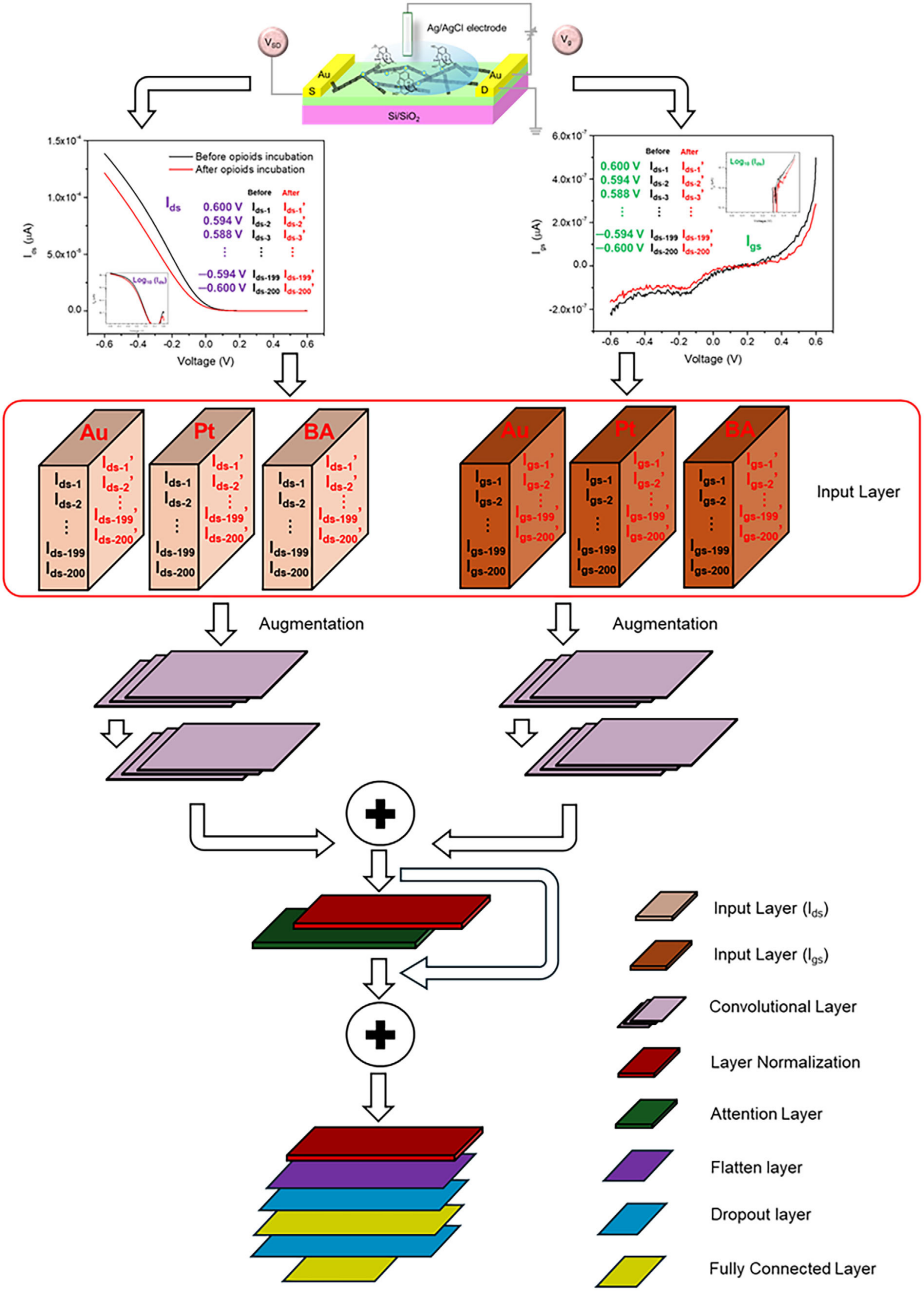
Flowchart of the deep learning task with CNN models incorporating both I_ds_ and I_gs_ values.

The deep learning task was realized by designing a multi‐path CNN model. The flowchart of the deep learning task with CNN model is shown in Figure 2. The input layers were composed of two input streams. One input stream included all the I_ds_ values at different gate voltages, and the other input stream included all the I_gs_ values. Since each training sample contained all the current values from all three types of sensors, there are 600 features in total (200 features for each sensor) in each input stream. The feature extraction was processed by two consecutive 1D convolutional layers. Finally, the flatten features passed through a fully connected layer with 128 neurons, and an output layer with four neurons provided the results. The dropout layer and Gaussian noise augmentation were introduced to improve the robustness of the model. The cross validation was based on a ″leave‐one‐sensor‐out method. More details about the CNN model were provided in the supporting information, and the model architecture was shown in Figure S4.

## 3 Results and Discussion

### 3.1 Sensor Fabrication: Characterization

**FIGURE 3:**
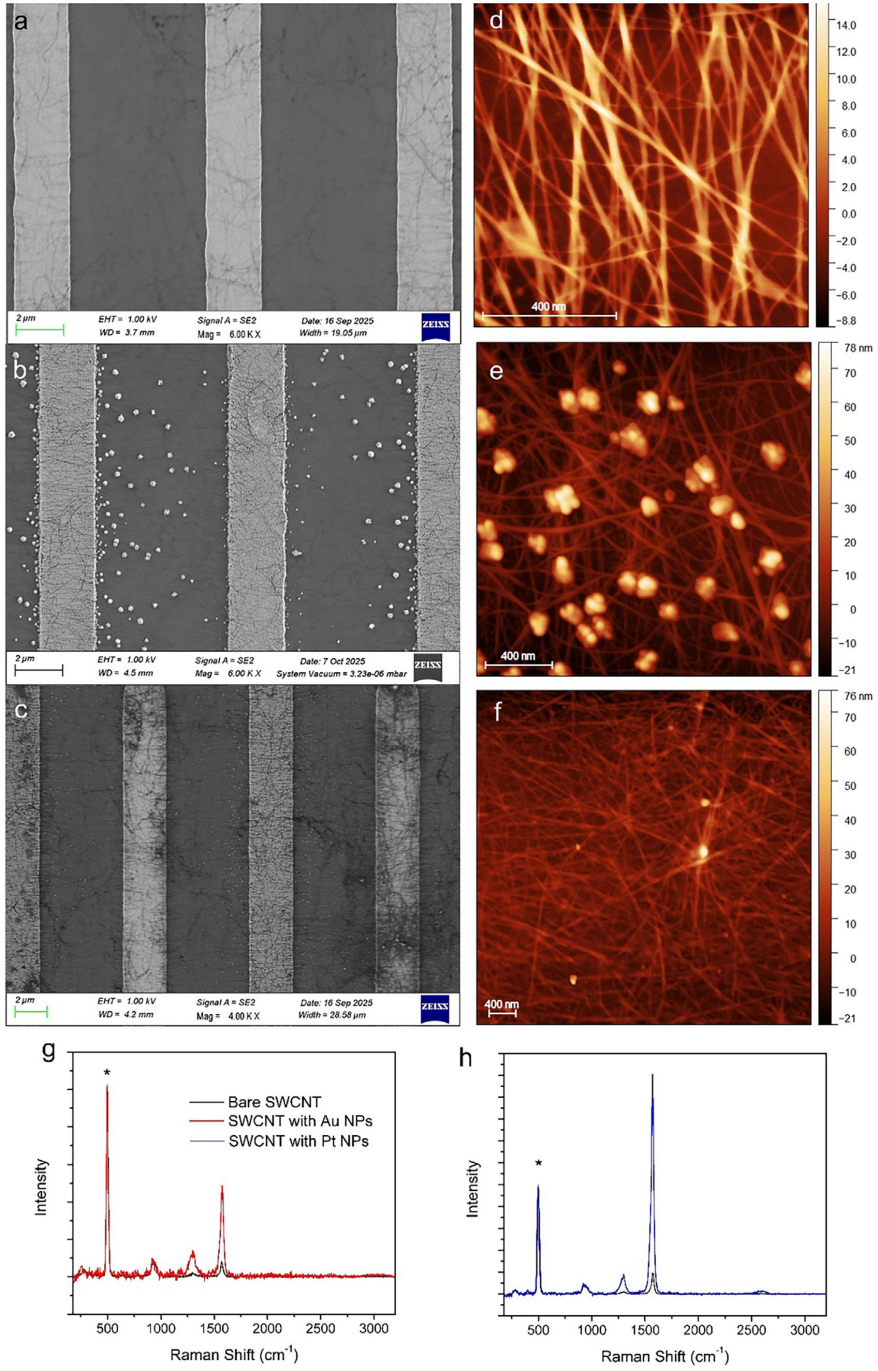
(a–c) SEM and (d–f) AFM images of (a,d) bare SWCNT and SWCNT decorated with (b,e) Au NPs and (c,f) Pt NPs. Raman spectra of bare SWCNT and SWCNT decorated with (g) Au NPs and (h) Pt NPs. All spectra were normalized to the Si peak labeled with asterisks at 499.8 nm.

The SEM image in Figure 3a shows the densely interconnected networks of SWCNT deposited on the silicon chip. The average diameter of bare SWCNT was 1.4 ± 0.5 nm, which was characterized with AFM (Figure 3d) and calculated with the height profiles of 20 different SWCNTs (Figure S5a and Table S2) on the AFM image. As shown in Figure 3b, gold nanoparticles (Au NPs) were then anchored on the bare SWCNT with an average diameter of 166.1 ± 81.8 nm (calculated by imageJ). Compared with Au NPs, platinum nanoparticles (Pt NPs) anchored on the bare SWCNT were smaller with an average diameter of 89.2 ± 31.6 nm (Figure 3c). The different sizes of two metal nanoparticles were also validated by AFM imaging, which showed that the decoration of Au NPs resulted in a height increase of 54.1 ± 13.9 nm (Figure S5b and Table S3) while only 37.3 ± 14.1 nm (Figure S5c and Table S4) for Pt NPs. It should be noted that both AuNPs and Pt NPs also grew on the interdigitated gold electrodes with smaller particle sizes (Figure S6a,b), and the aggregation of Au NPs could also be observed on both SEM and AFM images, likely due to homoaggregation of metal nanoparticles and heteroaggregation of metal nanoparticles with CNTs driven by van der Waals attractions [38, 39, 40]. To mitigate the influence of uneven particle sizes, the nanoparticle deposition strategy may be further optimized in future work, for example, by controlling particle size in the current electrochemical deposition process by optimizing the deposition voltage and salt concentration [41], or by physically depositing metal nanoparticles using pre‐made nanoparticle ink with a narrow size distribution. The decoration of metal nanoparticles was also demonstrated by the enhancement of D and G peaks intensity in the Raman spectra (Figure 3g,h) due to surface‐enhanced Raman scattering.

### 3.2 Sensor Fabrication: NTFET Characteristics on Different Sensors

Generally, metal nanoparticles decoration on bare SWCNT will result in an increase in I_ds_ due to the p‐doping of bare CNT, which is consistent with the statistical results of threshold voltage values (V_th_) of our 84 fabricated sensors shown in Figure S7a. Compared with the bare SWCNT, the SWCNT decorated with Au/Pt NPs shows a more positive V_th_, indicating that the bare CNT is p‐doped after metal nanoparticle decoration, which shifts the V_th_ more positive [42]. Meanwhile, the I_gs_ shows distinct behaviors after the decoration of metal nanoparticles, which increases dramatically by applying a more positive voltage (Figure S7b). One possible explanation for I_gs_ increase is the increase of capacitive current. It has been reported that the generation of I_gs_ on an electrolyte‐gated FET device is associated with both capacitive current and Faradic current [43, 44, 45], and electrode material may play an important role in the capacitive current due to its large contact area with electrolyte [43]. The decoration of metal nanoparticles, especially the growth of metal nanoparticles on the electrodes, may greatly expand the contact area between the electrolyte and the electrode, resulting in an increase of capacitive current. In addition, the I_gs_ values of Pt‐NTFET are obviously higher than the other two sensors, indicating that Faradic process may also contribute to the I_gs_ increase. The deposited metal nanoparticles may act as catalytic centers on the CNT interface, which catalyze the electrochemical reactions to generate Faradic current at high voltages. However, it should also be noted that the I_gs_ of bare CNT may be high (closed to 1 µA at 0.5 V), which is due to the lack of passivation of the interdigitated gold electrodes. In our experiments, the lack of electrode passivation is a compromise between the high sensitivity (the passivation of a single gold electrode may also block the whole channels nearby on the interdigitated electrodes area and lower the I_ds_) and suppression of gate leakage. Nevertheless, other effects can be taken to suppress I_gs_, such as the control of ionic strength to lower the capacitive current, and elimination of oxygen during FET sensor storage/testing to avoid Faradic process [44]. These results also demonstrate that I_gs_ provide crucial information for different sensors, which may be applied for further data training processes.

### 3.3 Opioid Sensing: NTFET Characteristics on Different Sensors

**FIGURE 4:**
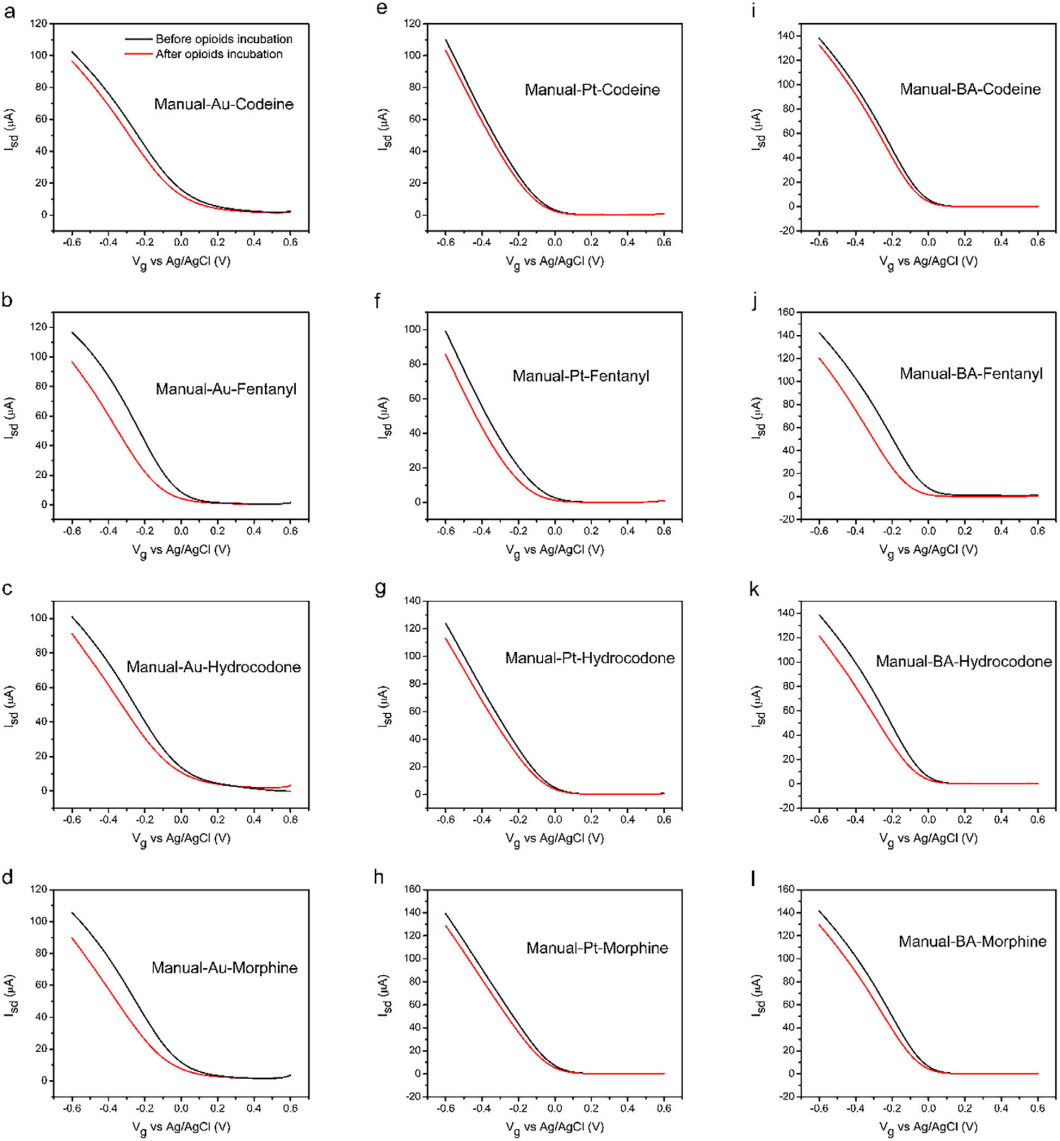
Transfer characteristics of NTFET, i.e, I_ds_ vs. applied liquid gate voltage, of differernt NTFET sensors after incubation with opioids. (a‐d) Transfer characteristics of Au‐NTFET after incubation with (a) codeine, (b) fentanyl, (c) hydrocodone, and (d) morphine, respectively. (e–h) Transfer characteristics of Pt‐NTFET after incubation with (e) codeine, (f) fentanyl, (g) hydrocodone, and (h) morphine, respectively. (i—l) Transfer characteristics of BA‐NTFET after incubation with (i) codeine, (j) fentanyl, (k) hydrocodone, and (l) morphine, respectively. Devices were incubated with phosphate buffer for 1 h as the control and opioids for another 1 h before the measurement of transfer characteristics. Drain current was measured by sweepting gate voltage (V_g_) from 0.6 to – 0.6 V. Source–drain bias voltage (V_sd_) was 50 mV. All the data was collected manually.

Fentanyl was chosen as one of the target analytes since the rapid increase in fentanyl overdose has been a health crisis in recent years. Codeine, hydrocodone, and morphine, other three compounds which also lead to public concerns about opioid abuse, were chosen as the other three analytes. These three compounds have similar structures but are different from fentanyl. The structures of these four opioid compounds are shown in Figure 1. Opioid molecules can be physically adsorbed onto the surfaces of metal nanoparticles. This property has been exploited for opioid detection using other techniques such as surface‐enhanced Raman scattering (SERS) [46, 47] and voltammetric methods [36], without the need for capture probe decoration. In addition, we have explored the classification of purine compounds in our previous work [33], and NTFET sensors decorated with gold (Au‐NTFET) and platinum nanoparticles (Pt‐NTFET) showed better performances in the classification. Therefore, Au‐NTFET and Pt‐NTFET were chosen for the construction of sensor array. Since the aromatic organic compounds could be non‐covalently attached to the sidewalls of SWCNT by *π*–*π* stacking [48, 49], the NTFET sensors only with bare SWCNTs (BA‐NTFET) were also applied in the sensor array. We first explored opioid sensing based on manual operations. Firstly, the NTFET characteristics after incubation of 1 µg of different opioids (10 µg mL^−1^, 100 µL) in PBS were evaluated. Figure 4 showed the transfer characteristic curves before and after opioid incubation. The changes in NTFET characteristics were first discussed by comparing I_ds_ changes, which were based on the discussion of change of I_ds_ at −0.5 V in Figure S8. Fentanyl led to the largest decrease in conductance after incubation. Compared with the other three compounds, fentanyl has more aromatic rings which may result in a strong interaction with the SWCNT sidewall by *π*–*π* stacking. In addition, metal nanoparticles showed higher affinity for fentanyl molecules, as validated by DFT calculations in our previous work [25]. For the other three, morphine and hydrocodone showed similar responses to the sensors, and codeine resulted in the lowest conductance changes. In addition to *π*–*π* stacking, polar‐*π* interactions between the hydroxyl groups of opioid molecules and the CNT sidewalls also contributed to adsorption of opioids on the sensor interface [50]. The relatively weak response to codeine may be attributed to steric hindrance from the 3‐methoxy group, which impedes interactions between codeine molecule and the CNT/nanoparticles. The nucleophilic and electrophilic centers of codeine also exhibit lower electron densities [51], which may further explain the weak interaction between codeine and the sensor interface. We then compared the conductance changes among different types of sensors. Compared with Pt‐NTFET and BA‐NTFET, Au‐NTFET showed higher response to all the opioids, indicating that the opioids might also have higher affinity to Au NPs on the interface with SWCNTs. Lastly, we investigated the stability of each sensor after reuse across tests of all four compounds. As shown in Figure S9, the I_ds_ at −0.6 V, which is typically the maximum current, remains essentially constant across all tests, with variances below 10% for all sensors. This demonstrates that the electrical performance of the FET sensors is well maintained after reuse and that sensors retain good stability upon storage.

### 3.4 Classification of Opioids in Manual Test Group With Conventional Supervised Learning Methods: Original Model With I_ds_ Only

The classification of opioids was first investigated using supervised learning methods. First, the models were trained based on the 15 features extracted from I_ds_ changes (original model in Table S1). Compared with the model developed in our previous publications [33, 34], less correlated features were employed for the model construction in this work, and several new features were added to discuss the change of electrical parameters missing out in our previous models, such as on‐off ratio and charge carrier mobility. The explanations of different features were detailed in Table S1. In addition, to eliminate the influence of Faradic processes at positive voltages, only I_ds_ values measured at V ≤ 0.3 V were used for feature extraction. As shown in Table S5, we tested different algorithms for model training, including LDA, support vector machine (SVM), k‐nearest neighbors (KNN), RF, and logistic regression (LR). LDA and RF were selected for the training of conventional supervised models in subsequent analyses. Subsequently, the model was first trained with data from only one type of sensor for the classification of the four compounds, while the results shown in Table S6 showed that the accuracy values were only 50% to 60% for all three types of sensors. Herein, we then explored the combination of data from all three types of sensors for model training, which generated 45 features (15 × 3) for model training. The combination of three sensors is strictly ruled. A given Au sensor could only be concatenated to another certain Pt/BA sensor for all four compounds, and free combination is forbidden. (In the opioid testing, the Au/Pt/BA sensor can be integrated on the same unit for the testing of all four compounds, so the concatenation of sensors is unique). The accuracy increased to 75.6% (LDA) for the manual test group. We still optimized the model with the recursive feature elimination method (RFECV) reported in our previous works [33, 34], and the four compounds can be classified with an accuracy value of 82.6% (LDA) using an optimized model with 18 features. Nevertheless, the classification of the four compounds based on the I_ds_ value should be further improved and other features may be introduced to improve model performance.

### 3.5 Classification of Opioids in Manual Test Group with Deep Learning Methods

Although the classification of opioids with conventional supervised learning algorithms could be improved with sensor combinations, there are only 15 features extracted from the transfer characteristics. However, manually assigning gate voltages and other parameters such as transconductance and V_th_ results in a “prejudiced” model. There are 200 data points in each data file, and most FET data is not effectively used for model training. Therefore, the FET data was further trained with a multi‐path CNN model, which utilized all the data values in a FET dataset. Besides the I_ds_ values, I_gs_ is also applied for data analysis. We have observed the difference of I_gs_ among different types of sensors after metal nanoparticle decoration. The interaction between opioid compounds and CNT/metal nanoparticles may further change the capacitive current by changing the contact area between CNT hybrids and electrolyte, and extra faradic current may be generated with the introduction of opioid compound, resulting in the change of I_gs_. Herein, I_gs_ values were also included in the CNN model, which introduced 200 more features for model training.

A deep learning model is commonly based on a large size of data. However, there are only limited samples in our experiment. To generate more samples used for CNN model training, the three types of sensors are allowed for free combination, which generates 43^3^ samples at most for the CNN model. In our model training, a “leave‐one‐sensor‐out” strategy is applied, which means that an Au/Pt/BA sensor is randomly picked out and combined as a “unknown” sample for validation with 1200 features (200 × 3 × 2, 200 features from each of 3 sensors and 2 channels for I_ds_ and I_gs_ separately), and the CNN model is trained with data based on the combination of remained sensor data (42^3^ at most). In each epoch, around 20 000 combined samples were randomly picked for model training and prediction of the picked‐out sample. The hyperparameters for the CNN model training were listed in Table S7.

The manual test group was trained for 38 epochs (training loss vs epoch curve is shown in Figure S10) and the accuracy for the manual group was greatly improved after the introduction of I_gs_ as features (98.3% vs 82.6%). Although the accuracy with CNN model is encouraging, it should be noted that the data amount is still limited and the model generality should be further investigated. Besides the model accuracy, what interests us more is the role of I_gs_ in the classification. We then evaluated the significance of each input stream in the final prediction by utilizing attention scores, which demonstrated the attribution of each input stream when the model was trained with both inputs. Both I_ds_ and I_gs_ pay similar attention to itself and the other one with an attention score of around 0.5 (Figure S11a,b), showing that both I_ds_ and I_gs_ play important roles in the model. Additionally, we evaluated the contributions of each of the 600 input features in I_ds_ and I_gs_ values. By integrating gradients from a baseline to the actual input, we pinpointed the most influential features driving our deep neural network's predictions. For each sample, the 20 most important features from I_ds_ and I_gs_ values were identified and categorized based on the sensor types, which were summarized in Table S8 based on the average of results from each sample. In general, all three types of sensors were closely involved in the classification of opioids. The difference is mainly the contribution of Pt‐NTFET in different tasks. Compared with another two sensors, Pt‐NTFET provides both least key I_ds_ and I_gs_ features. It's still unknown whether the fact is associated with the aforementioned distinct I_gs_ behavior of PT‐NTFET, which may be meaningful for further investigation. It should be noted that the contributions of different features vary dramatically across different samples, which are shown as the large deviations in Table S8. The evaluation of features demonstrated the key role of metal nanoparticles decoration and introduction of sensor arrays in the classification of opioids, which helped reveal more differences among different opioids and provided more information for the classification.

Compared with the conventional supervised learning model, the application of deep learning model provides new sights for the classification of opioids. The manual test group could be well classified with a high accuracy using the CNN model based on the free sensor combination, demonstrating it as a powerful tool for the classification of compounds with similar structures. More importantly, the CNN model clearly demonstrated the importance of I_gs_ in classification. Due to its low value, I_gs_ is often neglected in the electrolyte‐gated FET sensing tests and only I_ds_ is focused on. However, the CNN model reveals that I_gs_ is as important as I_ds_ in the classification of opioids, which can play a decisive role in the model and greatly improve its accuracy.

### 3.6 Improved Conventional Supervised Learning Model: Demonstrating the Significance of I_gs_

**FIGURE 5:**
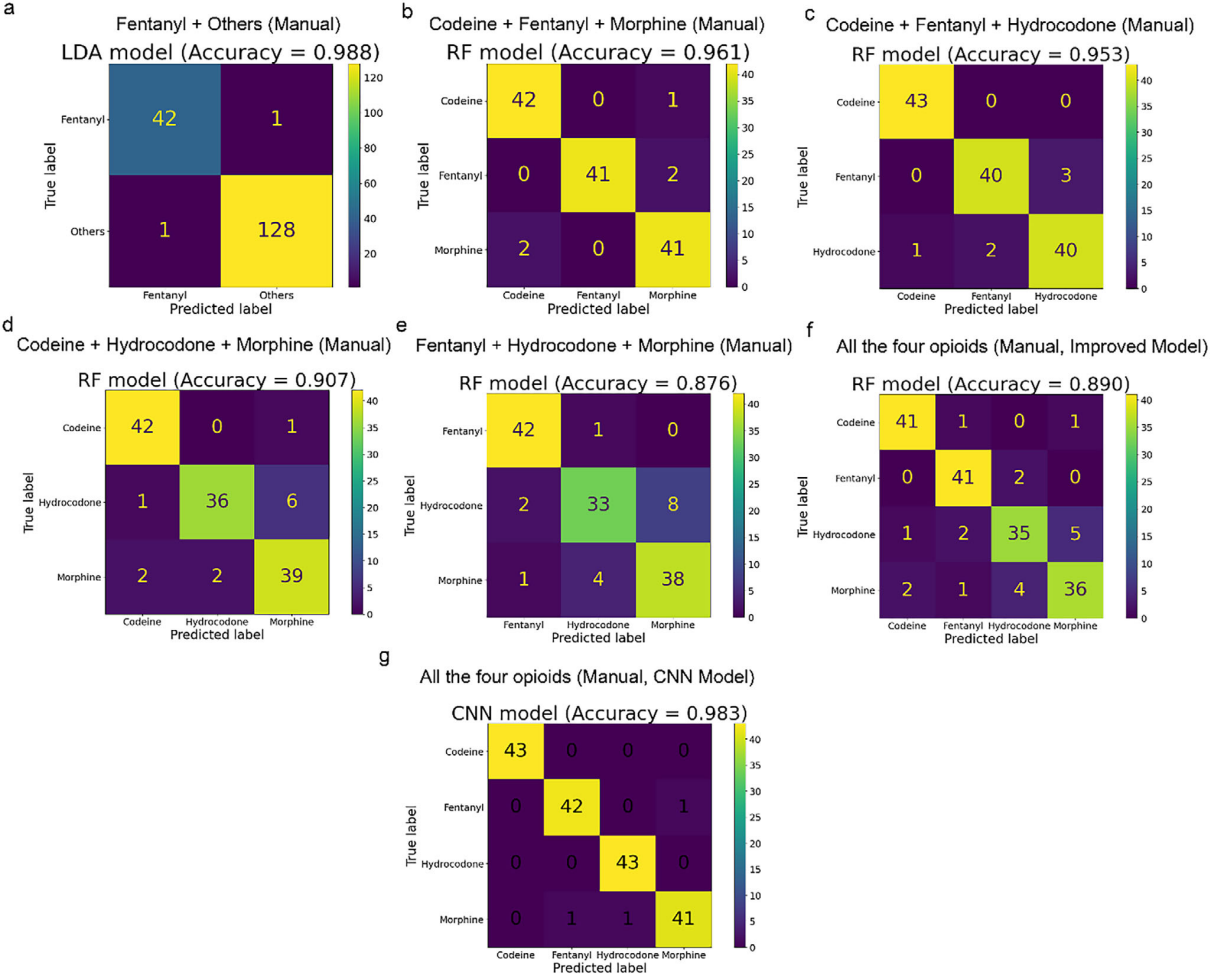
Confusion matrices for the manual test group, classified using conventional supervised learning models and the CNN model. (a‐f) Confusion matrices for different classification tasks: (a) fentanyl vs. other opioids, (b) codeine, fentanyl and morphine, (c) codeine, fentanyl and hydrocodone, (d) codeine, hydrocodone, and morphine, (e) fentanyl, hydrocodone, and morphine, and (f) codeine, fentanyl, hydrocodone, and morphine, using the improved conventional model with features extracted from both I_ds_ and I_gs_. (g) Confusion matrix for classification of all four opioids using the CNN model in the manual test group.

As the CNN model reveals that I_gs_ is crucial in the classification, we then explored whether the conventional model could be improved with the addition of I_gs_ feature. Herein, we built a new improved conventional supervised learning model (improved model in Table S1) by declining three I_ds_ features while adding four I_gs_ features to the model. Considering the high I_gs_ value at positive voltages, the I_gs_ features were extracted from I_gs_ values when gate voltage was lower than 0.3 V to avoid the influence of faradic process and inrush current when the transistor was turned on. Just like the original model, the sensor combination is still strictly ruled for model training with the improved model. As shown in Figure 5 and Table 1, the model accuracy increases to 89.0% for the manual group, supporting the importance of I_gs_ derived from the CNN model. The improved model was also used for the classification of less classes and satisfactory results were obtained. Fentanyl could be well distinguished from other compounds with an accuracy value of 98.8%. For the ternary classification tasks, the accuracy values range from 87.6% to 96.1% for the manual group. The classification of hydrocodone is the main challenge with the improved model. We also evaluate the importance of features (Table S9) with the RFECV method. For the I_ds_ features, six features including transconductance changes (F1), threshold shift (F2), maximum current changes (F3), and I_ds_ changes in the linear region (F9 to F11) are the most important features. The threshold shift and I_ds_ changes indicate the role of an electron doping mechanism in classification. The interaction between opioids and sensor interfaces results in n‐doping of CNT, which leads to the negative shift of V_th_ and I_ds_ decrease. Meanwhile, the change in transconductance reveals the involvement of Schottky barrier modulation, as the contact between CNT and metal electrodes/nanoparticles is influenced by the attachment of opioid molecules to the sensor interface. The influence of metal‐CNT contact is also reflected in changes of I_gs_. I_gs_ features also play a key role in classification, and three of the four I_gs_ features appear in most optimized models. Especially, I_gs_ change at 0.3 V is most significant and participates in all the optimized models. The importance of I_gs_ derived from the improved model is consistent with the CNN model. Compared with the original model, the improved model also has even less correlated features (Figure S12). Our results indicate that the conclusion derived from CNN models offers guidance to improve the conventional models successfully, which effectively lowers the influence of noise and improves prediction accuracy.

**TABLE 1:** Classification of different opioids using the improved coventional supervised learning model after optimization with RFECV method. The leave‐one‐out method was applied for cross‐validation.

	Fentanyl and others	Codeine, Fentanyl, and Morphine	Codeine, Fentanyl, and Hydrocodone	Codeine, Hydrocodone, and Morphine	Fentanyl, Hydrocodone, and Morphine	Codeine, Fentanyl, Hydrocodone, and Morphine
Manual Test Group						
Accuracy	0.988	0.961	0.953	0.907	0.876	0.890
Selected Algorithm	LDA	RF	RF	RF	RF	RF
Number of Features	33	20	26	48	41	32

Automated Test Group						
Accuracy	0.976	0.919	0.870	0.780	0.805	0.805
Selected Algorithm	LDA	RF	LDA	LDA	LDA	LDA
Number of Features	26	12	20	27	22	22

### 3.7 Classification of Opioids With the Automated Testing System

For opioid sensing tests, numerous manual operations should always be repeated, which are tedious and not favorable for the batch testing of different opioids in routine applications. Herein, we then explored opioid sensing with our developed automated electrolyte‐gated FET test system, which could realize the testing of up to 96 different FET sensors in a task [37]. The architecture of the automated system and its workflow are discussed in detail in another work [37].

As shown in Figure S3, a similar protocol was applied to the automated system with some differences in the volume of washing buffer and gas drying process. The NTFET characteristics after opioid incubation are plotted in Figure S13, and the changes of I_ds_ at −0.5 V are shown in Figure S14. Compared with the manual test group, we observe different I_ds_ changes of BA‐NTFET and Pt‐NTFET in the automated test group. Pt‐NTFET shows larger I_ds_ changes in the manual test group while BA‐NTFET results in larger I_ds_ changes in the automated test group. We originally expected that the automated test system could standardize the experimental operations and eliminate the random errors due to the manual operations, while the results actually show that new testing errors, at least systematic errors, are introduced with the automated system. An important reason may be the lack of gas drying process. We found that the residual liquid might form a liquid membrane on the chip surface due to the surface tension. Without the gas drying process, the residual liquid could not be thoroughly removed by the pipette, the residual analyte/rinsing buffer may continuously interact with CNT hybrids for longer time and potentially affect the electrolyte‐gated FET testing. We also explored testing using the automated system with an additional gas drying step. Ten Au‐NTFET sensors were used for fentanyl testing with the automated system. After the rinsing process was complete, the automated system was paused, and the sensors were dried manually with nitrogen gas prior to electrical measurements. The same ten sensors were also tested manually on the following day after storage. As shown in Figure S15, the FET test results from the automated system closely match those from the manual tests, with similar mean values and RSD values in the statistical analysis. These results demonstrate that the influence of the missing gas drying step in the automated protocol can be effectively alleviated by adding a manual drying step. However, it should be noted that systematic errors between the two protocols still exist, and these may also be associated with other protocol differences, such as differences in electrical measuring instruments, reference electrodes, and solution volume. Since a fully automated protocol without any manual intervention is the ultimate goal, model training in the automated group will continue to be based on data collected without the gas drying step.

The data collected by the automated system was then trained with both the conventional models and the CNN model. The original model could only realize an accuracy value of 72.5% for the automated test group. As shown in Table S1, compared with the improved model used for the manual group, the I_ds_ change at –0.1 V was included in the improved model for the automated group as it may play a more important role in the classification than I_ds_ change at −0.3 V. Using the improved model with RFECV method, the accuracy increases to 80.5% for the classification of four compounds, and 78.1% to 91.9% for the ternary classification tasks (Figure S16 and Table S11). Besides the generally lower accuracy compared with the manual test group, the automated test group also shows other differences. The LDA model shows higher accuracy for the automated group while the RF model matches the manual group better. The I_ds_ features still show a similar trend in the improved model for the automated group. While compared with their key roles in the manual group, I_gs_ features are much less important in the automated group. The results indicate that the data collected with the automated system is distinct from the manually collected data, which shows even greater differences in the CNN model. The accuracy unexpectedly decreased to 68.7% for the automated test group predicted by the CNN model. A classification report comparing the performance of the original model, the improved model, and the CNN model for both the manual and the automated groups, including accuracy, precision, recall, and F1 scores, is shown in Table S12. I_ds_ features also have a slightly higher self‐attention score than I_gs_ in the automated group (Figures S11c,d). Especially, I_gs_ allocates about 59.6% of its attention to I_ds_ while only dedicates 40.4% to itself on one attention head. In addition, Pt‐NTFET still shows distinct behaviors in feature importance (Table S10), which provides most important I_ds_ features while least important I_gs_ features.

The inconsistency between the manual/automated test groups with the CNN model reveals limitations of both our automated system and machine learning models. We first considered whether the free sensor combination strategy might affect model performance. Accordingly, we also evaluated the CNN model using only validation samples composed according to physical rules—the same combinations used in conventional models. As shown in Table S13, we systematically tested different learning rates and dropout rates, and CNN models were trained for approximately 100 epochs. The accuracy values were 91% for the manual group and 66% for the automated group, indicating that the free combination strategy may result in a slight overestimation or underestimation of model performance. Nevertheless, the test results based on ruled combinations remain close to the previously optimized CNN model, demonstrating that different sensor combinations do not lead to dramatic fluctuations in accuracy and that the CNN model is robust. However, it alone does not fully explain the accuracy gap between the two groups. We should admit that the shortages of our developed automated system, the drawbacks still existing in the operations of the automated system actually introduce new testing errors and bring new data quality issues, and these shortages are further maximized by the CNN model. The training loss curves show that the automated system introduces higher levels of systematic variability into FET measurements compared with the manual protocol. As shown in Figure S10, the manual group maintains a lower training loss, indicating that the model converges to confident predictions. In contrast, the higher loss across the training in the automated group shows the model could not realize the same confidence level as the manual group, resulting in a lower signal‐to‐noise ratio in the automated group. CNN models are inherently more sensitive to this systematic variability than conventional models, as they are trained with raw data directly and are therefore susceptible to fitting noise patterns as well as the true discriminative features, which degrades classification performance when the noise is structured and systematic. We also built two additional CNN models with different architectures (Figures S17 and S18) and obtained similar model performance (additional Model A: 90% for the manual group and 67.3% for the automated group; additional Model B: 88% for the manual group and 68.3% for the automated group), supporting the conclusion that the drop in accuracy in the automated group was attributable to data quality resulting from the introduction of the automated system, rather than to the CNN architecture. On the other hand, the low accuracy also indicates that the CNN model is not generalized. Due to the significant differences between the data in the two groups, a single CNN model may not be applied for the classification of opioids tested through different approaches, and different models should be optimized separately for each group. In routine applications, the testing results may vary with different factors such as sample matrices, storage conditions, and testing days, and a generalized model should be developed to fulfill the screening of plenty of samples. Otherwise, it's also possible that robot data is more objective, and the operator's bias in manual operations may result in an overestimation of accuracy in the manual test group. Lastly, the device‐to‐device variation may also contribute to the inconsistency. The large error bars in Figures S8 and S14 indicate that the device‐to‐device variation is large, and the feature values are widely distributed. Device‐to‐device variations may originate from different steps in sensor fabrication process, including the unidirectional alignment of CNT and the aggregation of metal nanoparticles. In addition to the I_gs_ changes discussed above, nanoparticle aggregation may also contribute to the distribution of V_th_ and I_ds_ current increase [52, 53]. The large deviations in feature values may result in low data reliability, which introduces more noise and affects model performance.

## 4 Conclusions

In this work, a NTFET sensor array was constructed for the classification of one microgram of opioids with machine learning. We explored the classification of four different opioids including codeine, fentanyl, hydrocodone, and morphine with machine learning methods based on both conventional supervised learning model and deep learning model. The introduction of CNN model realized impressive model performance for the manual test group with an accuracy of 98.3%. More importantly, the CNN model revealed the importance of I_gs_ in the classification and successfully advised the design of improved conventional supervised learning model. The constructed model was also applied for binary/ternary classification tasks and satisfactory results were obtained.

The demonstrated application of machine learning methods extends beyond traditional classification tasks based on a predefined set of FET parameters derived from our current understanding of sensor mechanisms. By feeding raw sensor data directly into the models and allowing for data‐driven optimization, we can uncover hidden or previously unknown sensing mechanisms. This “unprejudiced” approach enables evaluation of the relative importance of various device characteristics in distinguishing analytes based on their molecular interactions with the sensor surface.

However, we should also admit a series of limitations of our approach. Compared with other methods for the quantitative analysis of opioids, a compromise in the concentration of analytes was made for data collection, which might limit its application at low concentration level. Due to the lack of capture probes such as opioid antibodies, our approach has no selectivity to different opioids and results in lower accuracy. In addition, all the tests were done in the phosphate buffer while the opioids screening in complex matrices such as human body fluids is also important. In this work, we also explore the opioid sensing with the automated electrolyte‐gated FET testing system, while the discrepancy between two groups indicates the shortages in the model generalization, which requires both high data quality and more data for model training. It also reminds us that the drawbacks of our developed automated system and deviations in sensor fabrication. Although limitations remain, we believe this proof‐of‐concept work can open opportunities for future development of a sensor array for the detection of various opioid drugs at lower concentration level and in more complex media. Additionally, we will continue optimizing the sensor fabrication protocol and improving the automated system to reduce the device‐to‐device variations, such as introducing a drying module into the automated system and automating the SWCNT deposition process by material printing. An additional batch calibration step may also be essential in sensor fabrication for quality control, to reduce tolerances and discard non‐qualifying devices. More data will also be collected for model training, and the model will be further optimized to improve its robustness and accuracy.

## Conflicts of Interest

The authors declare no conflicts of interest

## Supporting information




**Supporting File**: smll74244‐sup‐0001‐SuppMat.pdf.

## Data Availability

The data that support the findings of this study are available from the corresponding author upon reasonable request.
