# Supplementary material for: Nanoelectronic Detection of Opioids: Machine Learning‐Powered Screening With Carbon Nanotube Field‐Effect Transistor Sensor Array
Source: Small. 2026 Jun 19;22(49):e74244. doi: 10.1002/smll.74244 (PMC13533242; doi:10.1002/smll.74244)
Supplement: Supplementary file 1 [file SMLL-22-e74244-s001.pdf]

Supporting Information

Nanoelectronic Detection of Opioids: Machine Learning-Powered Screening with Carbon Nanotube Field-Effect Transistor Sensor Array

Zhengru Liu,[†] *Mehdy Dousty,*[‡] *Wenting Shao,*[†] *Jieyu Wang,*[†] *Samia Afrin,*[†] *Wuqi Li,*[‡] *Ervin Sejdić,*[‡ §] *and Alexander Star*[† ||]*

[†]Department of Chemistry, University of Pittsburgh, Pittsburgh, Pennsylvania 15260, USA

[‡]The Edward S. Rogers Department of Electrical and Computer Engineering, University of Toronto, Toronto, Ontario M5S, Canada

[§]North York General Hospital, Toronto, Ontario M2K 1E1, Canada

[||]Department of Bioengineering, University of Pittsburgh, Pittsburgh, Pennsylvania 15261, USA

Correspondence to: astar@pitt.edu

Table of Contents

Methods……………………………………………………………………………………**S-4**

Figure S1. The integrated electrodes deposited with carbon nanotubes. **S-7**

Figure S2. Test protocol in opioids sensing with the sensor array for manual test group. ... **S-8**

Figure S3. Comparison of workflow between manual test group and automated test group in the opioid sensing tests. **S-9**

Figure S4. Architecture of the multi-path convolutional neural network. **S-10**

Figure S5. Atomic force microscopy (AFM) images of NTFET devices. **S-11**

Figure S6. SEM image of (a) gold and (b) platinum nanoparticles grown on the electrode materials. **S-11**

Figure S7. The statistics of (a) threshold voltage (V_{th}) and (b) gate-source current (I_{gs}) of the fabricated sensors. **S-12**

Figure S8. The changes of source-drain current at −0.5 V after incubation with different opioids on each type of sensor. All the data was collected manually. **S-12**

Figure S9. Stability of sensors after reuse. The evaluation is based on (a) the 43 sensors in the manual group and (b) the 41 sensors in the automated group. **S-13**

Figure S10. The training loss verse epoch curve of (A) manual and (B) automated test group using the CNN model. **S-13**

Figure S11. Attention scores of each input stream in the (a, b) manual and (c, d) automated test group on the two attention heads. **S-14**

Figure S12. The Pearson correlation coefficient values between two different features in (a) the (a) original and (b) the improved conventional supervised learning model. The fentanyl sensing data collected by Au-NTFET was applied for plotting. **S-15**

Figure S13. Transfer characteristics of NTFET, i.e, source-drain current (I_{sd}) versus applied liquid gate voltage, of differernt NTFET sensors after incubation with opioids. All the data was collected with the automated electrolyte-gated FET test system. **S-16**

Figure S14. The changes of source-drain current at −0.5 V after incubation with different opioids on each type of sensor. All the data was collected with the automated electrolyte-gated FET test system. **S-18**

Figure S15. Changes in source-drain current at −0.5 V after incubation with fentanyl using (a) 10 different Au-NTFET sensors and (b) statistical results. Each sensor was first tested using the automated system with an additional manual drying step, then tested again manually on the following day. **S-18**

Figure S16. Confusion matrices for the automated test group, classified using conventional supervised learning models and the CNN model. **S-19**

Figure S17. Architecture of the additional CNN Model A. In this model, the receptive field is increased to incorporate more global information during training. **S-20**

Figure S18. (a) Architecture of the additional CNN Model B, with the details of (b) Stream 1 and (c) Stream 2. In this model, superposition is assumed to occur in the raw signal domain rather than in the latent space. **S-21**

Table S1. The different features in the original and improved conventional supervised learning models. **S-22**

Table S2. Height profiles labeled in Figure S5a for the calculation of the diameter of bare SWCNT. **S-24**

Table S3. Height profiles labeled in Figure S5b for the calculation of the diameter of gold nanoparticles. **S-25**

Table S4. Height profiles labeled in Figure S5c for the calculation of the diameter of platinum nanoparticles. **S-26**

Table S5. Classification of four opioids in both the manual group and the automated group using different algorithms and all available features. **S-27**

Table S6. Classification of four opioids in the manual test group based on different combinations of sensors using the original conventional supervise learning model. **S-27**

Table S7. Hyperparameter grid search results for the CNN model. **S-28**

Table S8. The twenty most important I_{ds} and I_{gs} features categorized by sensor types for the classification of different opioids in the manual test group. The numbers were calculated based on the average results from each sample. **S-29**

Table S9. Features remained in the improved coventional supervised learning models after elimination with RFECV method. All the data was manually collected. The leave-one-out method was applied for cross-validation. **S-30**

Table S10. The twenty most important I_{ds} and I_{gs} features categorized by sensor types for the classification of different opioids in the automated test group. The numbers were calculated based on the average results from each sample. **S-31**

Table S11. Features remained in the improved coventional supervised learning models after elimination with RFECV method. All the data was collected with the automated system. The leave-one-out method was applied for cross-validation. **.. S-32**

Table S12. Classification report for the original model, improved model, and CNN model for both the manual group and the automated group. **.. S-33**

Table S13. Classification results for the manual group and the automated group using the CNN model validated on the same sensor combinations as the conventional models. **................. S-35**

Appendix A. Python Codes for the Classification of Opioids with Leave-one-out Cross-Validation. **... S-36**

Appendix B. Python Codes for the Opioid Sensing with an Automated Electrolyte-gated FET Test System. **... S-37**

Methods

Multi-Path Convolutional Neural Network. We implemented a multi-path convolutional neural network designed to process two distinct input streams. Each stream underwent separate feature extraction steps followed by feature fusion and classification.

Input Layers. The architecture utilized two independent input streams (Input1 and Input2) to receive input data. One input included I_{ds} values and the other included I_{gs} values, representing different data modalities. Each stream was processed independently through dedicated layers for feature extraction.

Feature Extraction. Each input stream was processed by third consecutive 1D convolutional layers. This third-layer setup captured hierarchical spatial features and ensured efficient feature representation.

First Convolutional Layer: Applied a 1D convolution operation with 128 filters and a kernel size of 2, followed by a rectified linear unit (ReLU) activation function.

Second Convolutional Layer: Used a 1D convolution operation with 128 filters for the manual group and 64 filters for the automated group, as well as a kernel size of 1, followed by a ReLU activation function.

Third Convolutional Layer: Used a 1D convolution operation with 128 filters for the manual group and 256 filters for the automated group, as well as a kernel size of 1, followed by a ReLU activation function.

Feature Fusion and Shared Layers. The outputs from the two input streams were concatenated along the channel axis to form a unified feature representation. The concatenated features were then processed with an attention layer with and layer normalization.

Dense Layers and Output Layers

Dense Layer: The flatten features passed through a dense layer with 128 neurons and a ReLU activation function, capturing global representations.

Output Layer: A final dense layer with 4 neurons provided multi-class classification outputs.

Regularization and Augmentation

Dropout: Dropout layers with a dropout rate of 0.4 were added after the dense layers to prevent overfitting.

Gaussian Noise Augmentation: Gaussian noise with a variance in the range of [0, 0.5] was optionally added during training to enhance the model's robustness.

The model was trained using the Adam optimizer with a cosine decay learning rate schedule with a starting learning rate of 0.01. The model was trained for 38 epochs for the manual group and 29 epochs for the automated group with a batch size of 4096 and was used to process the data efficiently. A random sampling strategy was employed to create training data. Three random indices were selected from the training pool. If the labels for these indices match across all input streams, their data were combined into a single training sample and appended to Train Data. Two inputs to the model are based on the concatenation of three sensors (Au-NTFET, Pt-NTFET, and BA-NTFET) along Y and Z channels. Augmented samples were added to the training data in which the random input channels are replaced with zero value. This introduced noise and increases the model's robustness by simulating missing or corrupted data. Mean and standard deviation were calculated for the training set and applied to normalize both training and validation data. In addition, an extensive hyperparameter search was performed and examined hyperparameters are shown in Table S6. We reported on the selected hyperparameters.

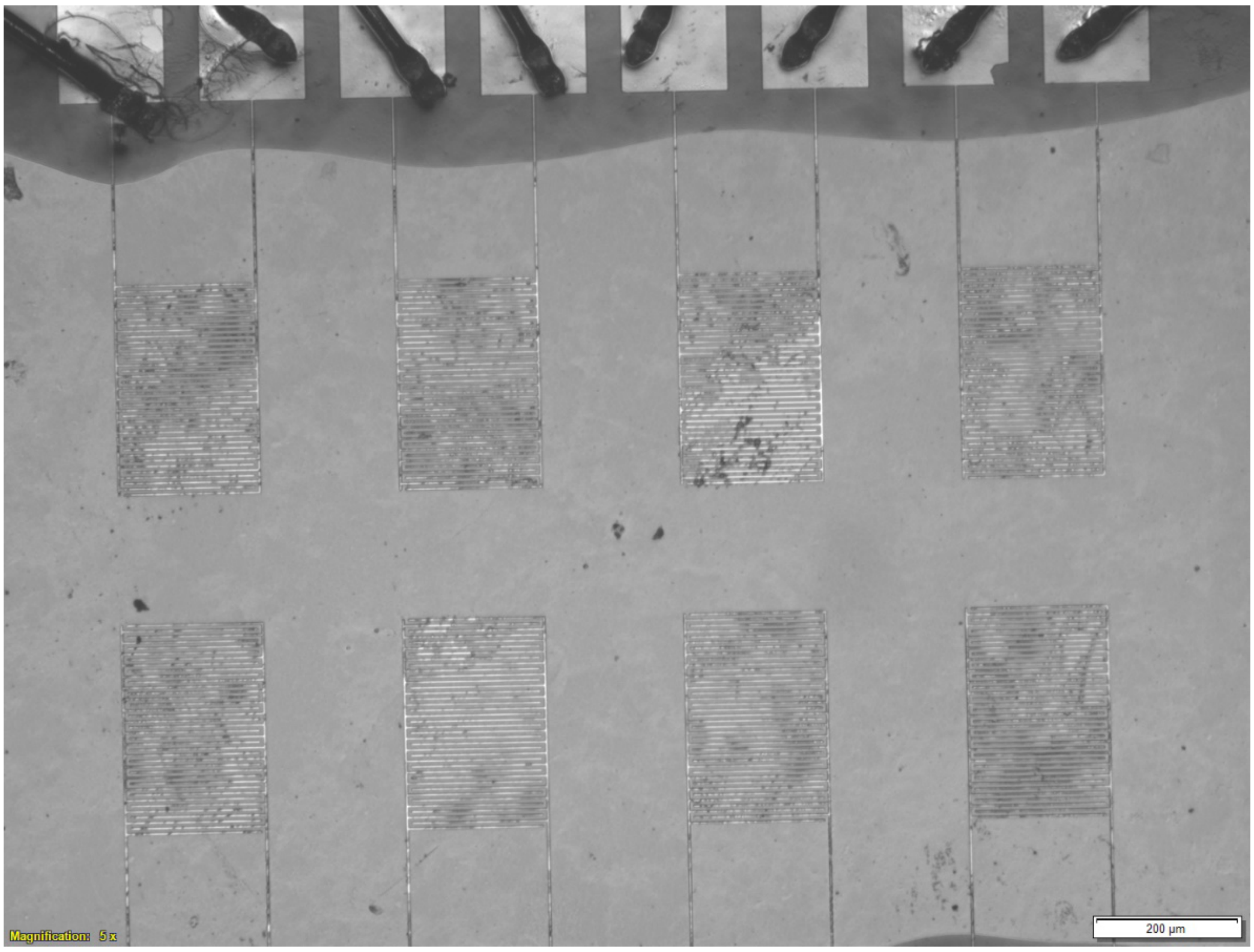

Figure S1. The integrated electrodes deposited with carbon nanotubes.

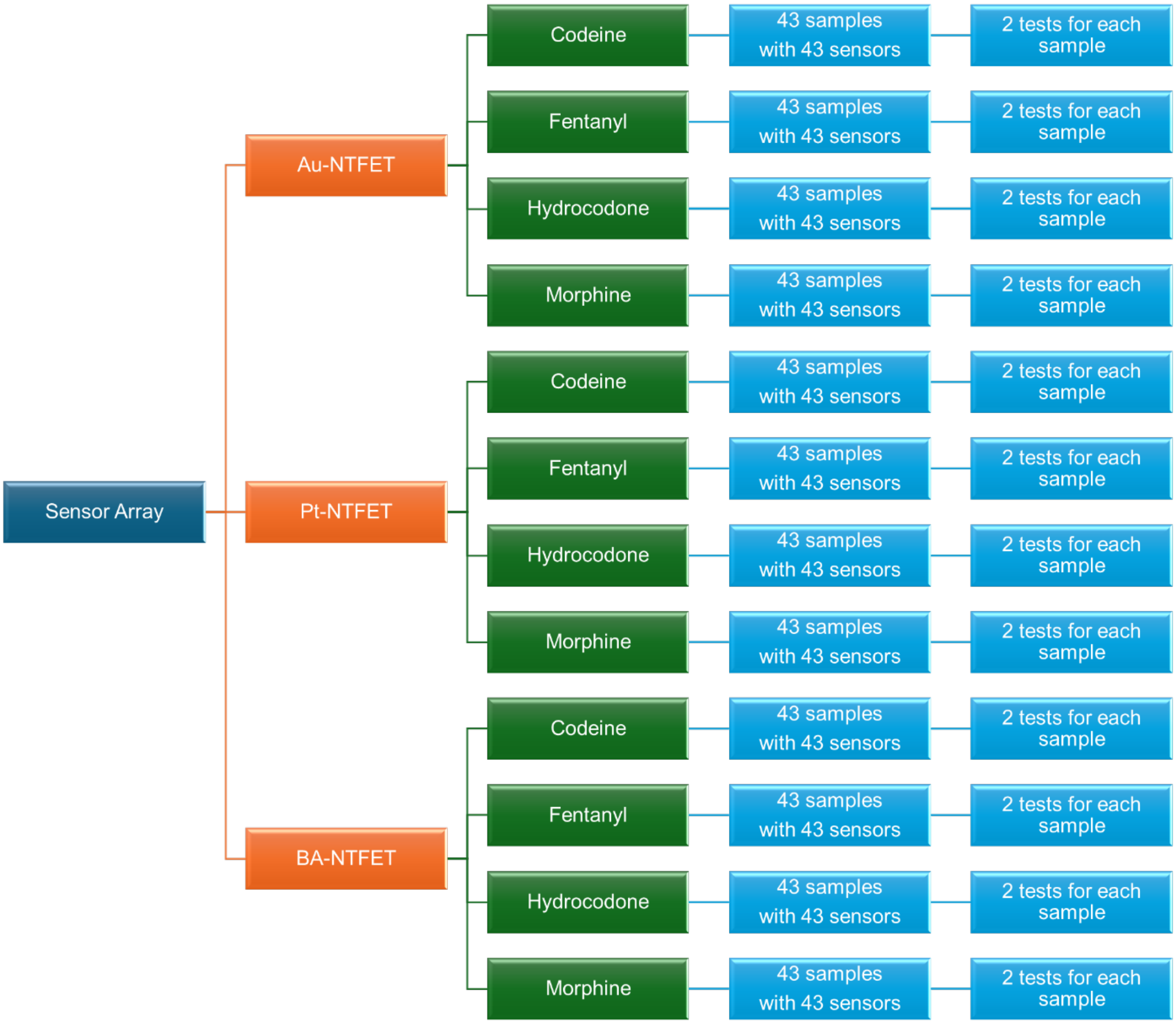

Figure S2. Test protocol in opioids sensing with the sensor array for manual test group.

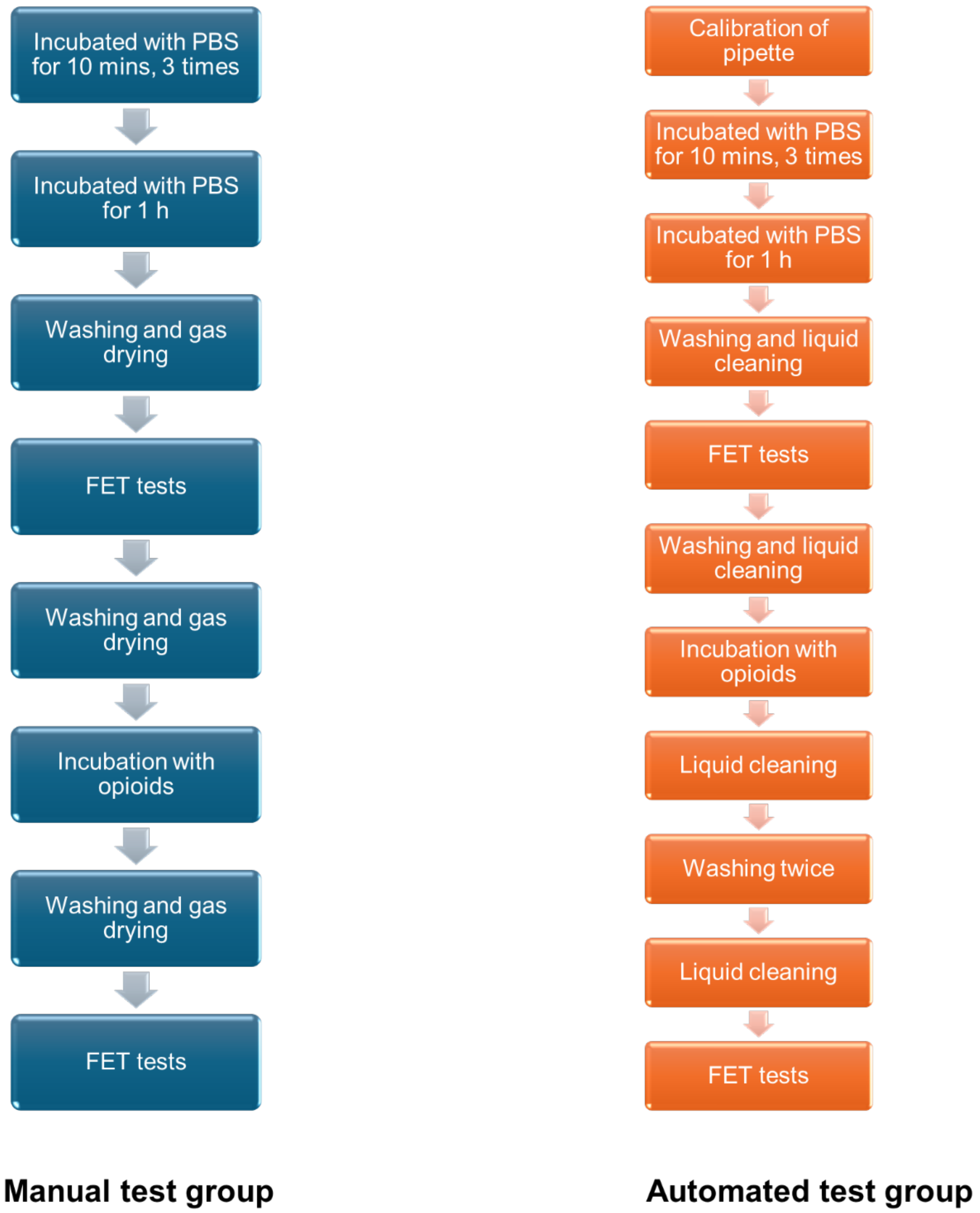

Figure S3. Comparison of workflow between manual test group and automated test group in the opioid sensing tests.

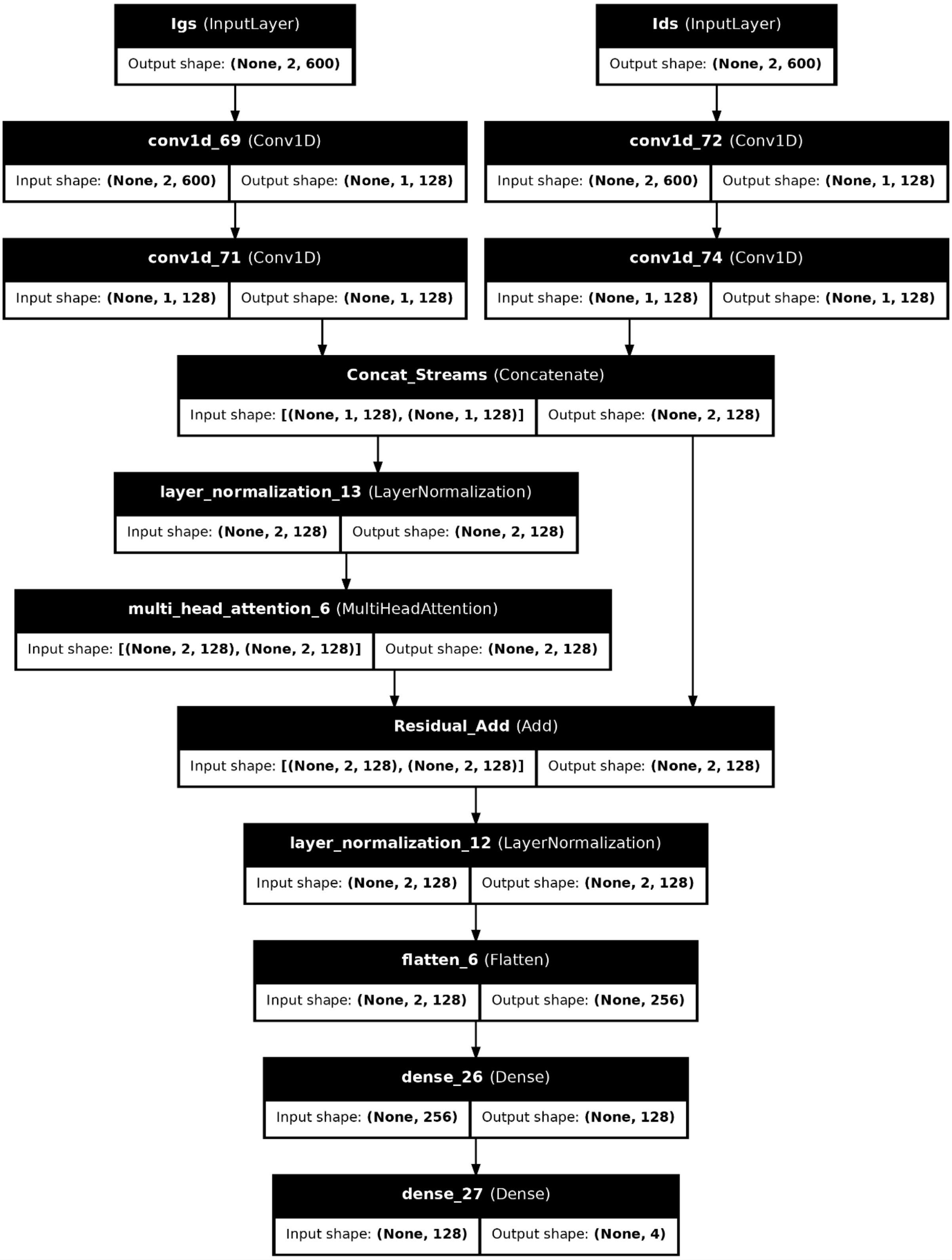

Figure S4. Architecture of the multi-path convolutional neural network.

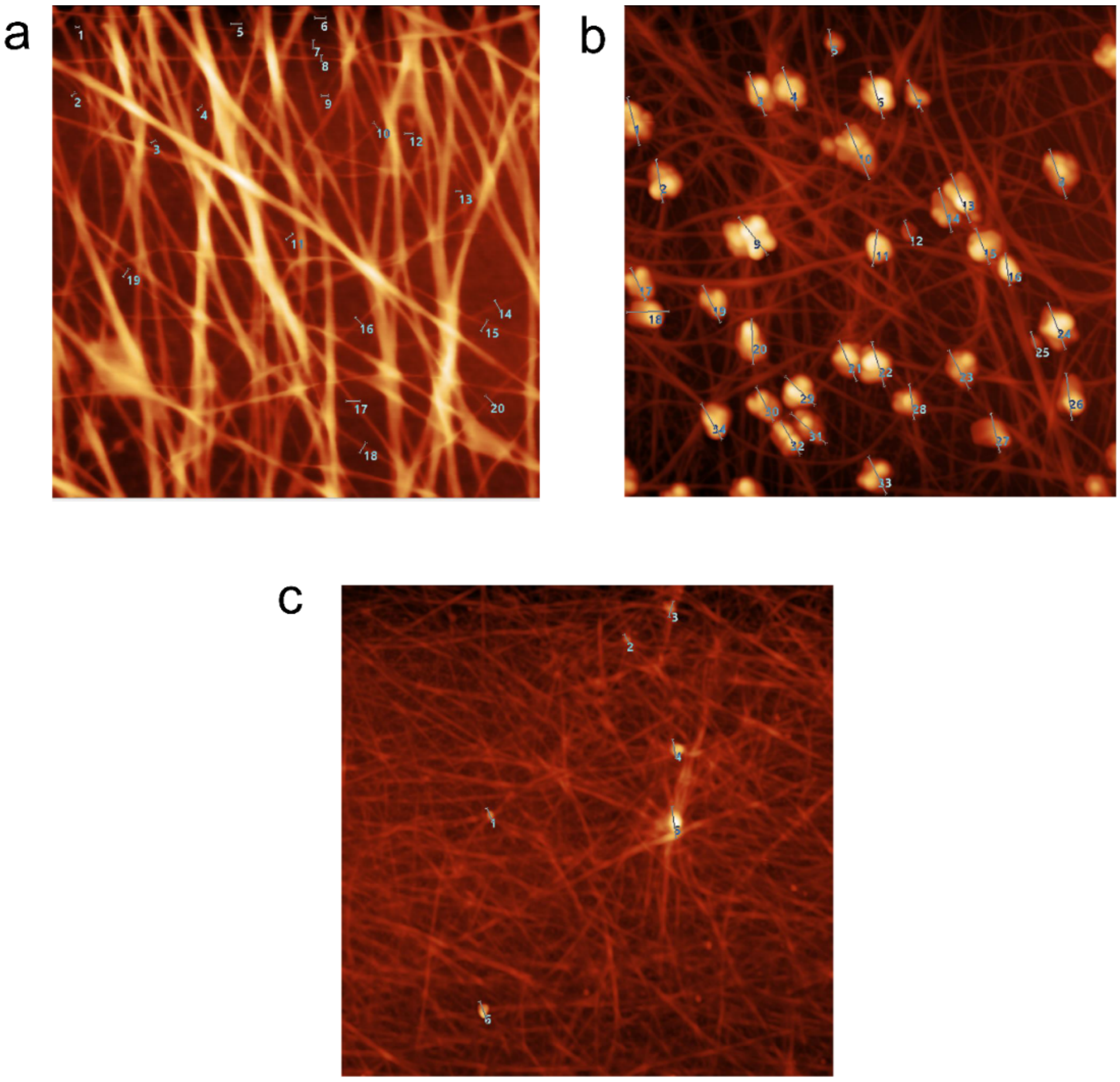

Figure S5. Atomic force microscopy (AFM) images of NTFET devices. (a) Bare SWCNT and SWCNT decorated with (b) gold and (c) platinum nanoparticles. For calculation of the diameter, the height of each labeled line segment was obtained by cacluating the difference between maximum and minimum value in each height file (Tables S2 to S4).

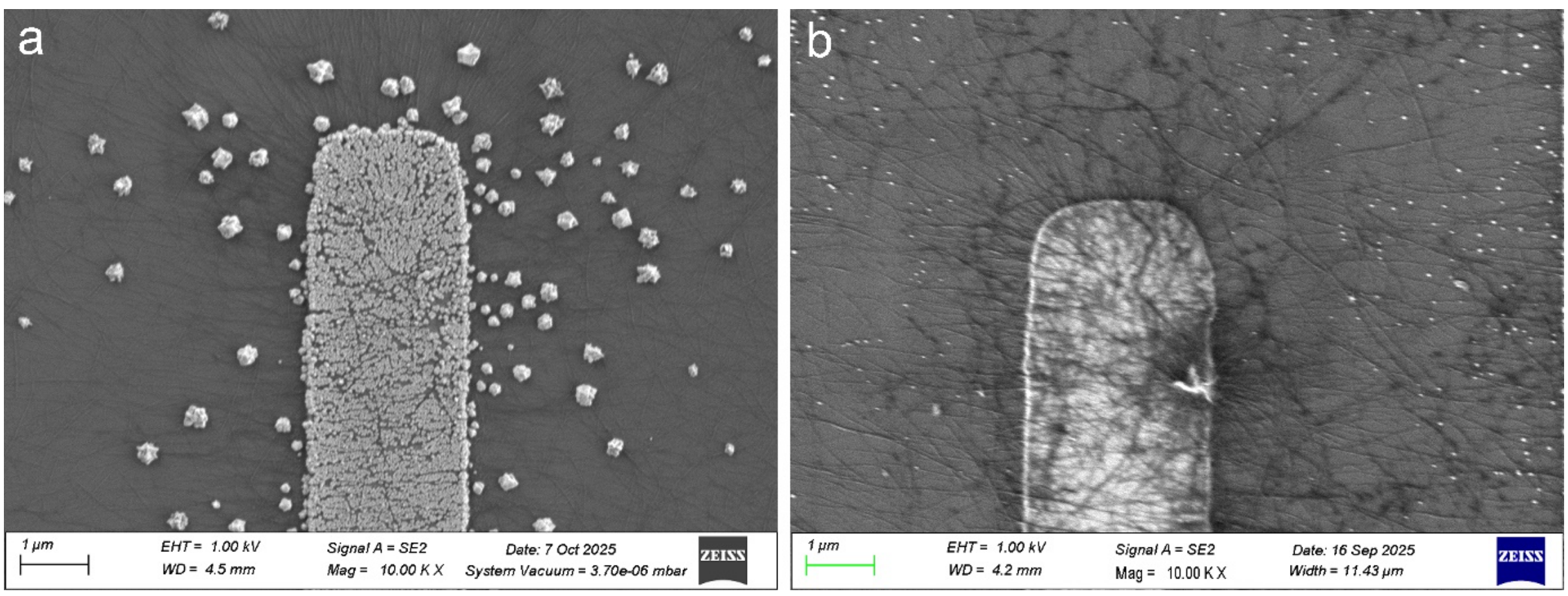

Figure S6. SEM image of (a) gold and (b) platinum nanoparticles grown on the electrode materials.

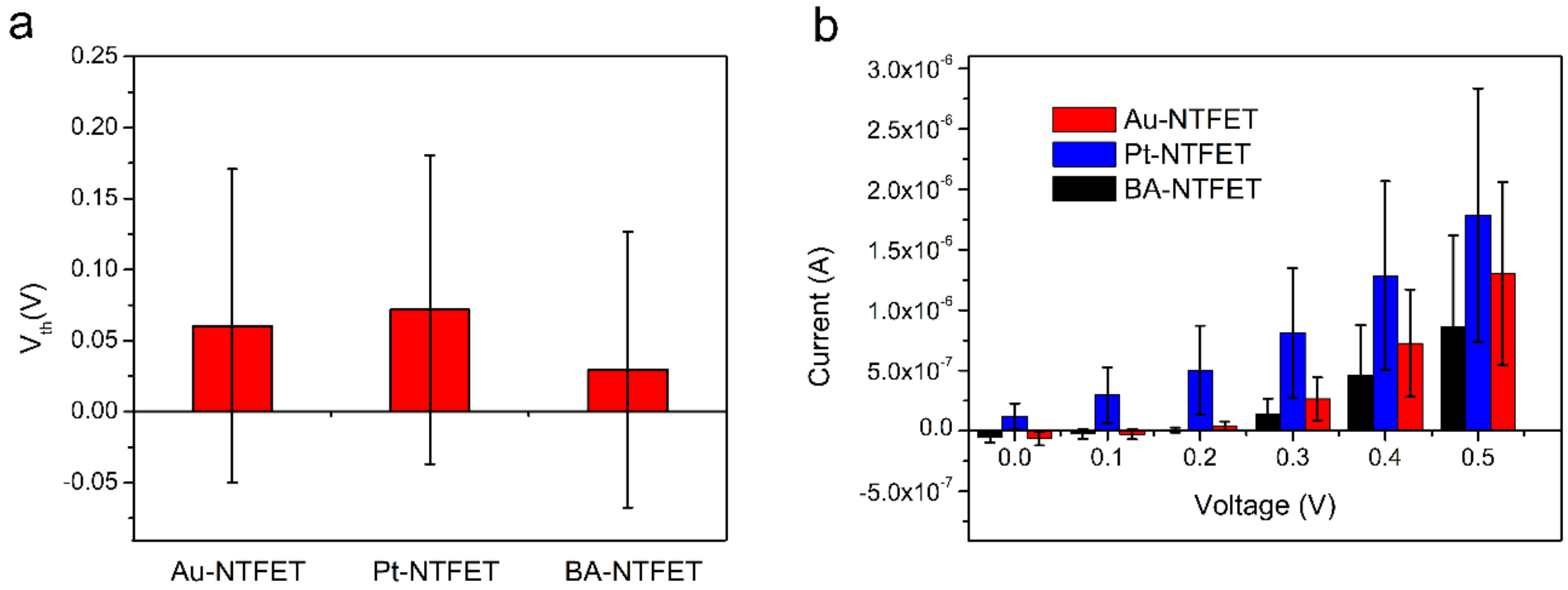

Figure S7. The statistics of (a) threshold voltage (V_{th}) and (b) gate-source current (I_{gs}) of the fabricated sensors.

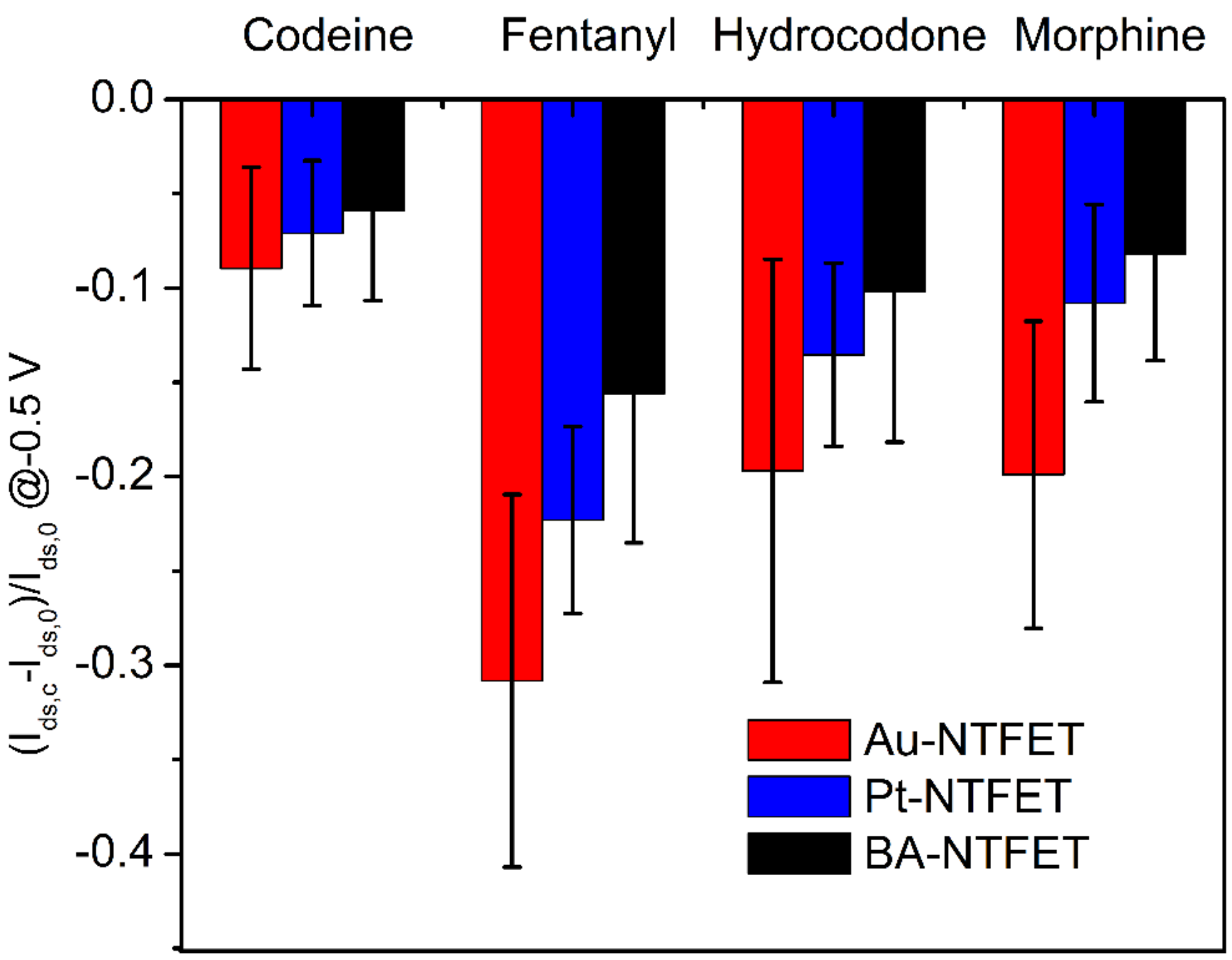

Figure S8. The changes of source-drain current at −0.5 V after incubation with different opioids on each type of sensor. All the data was collected manually.

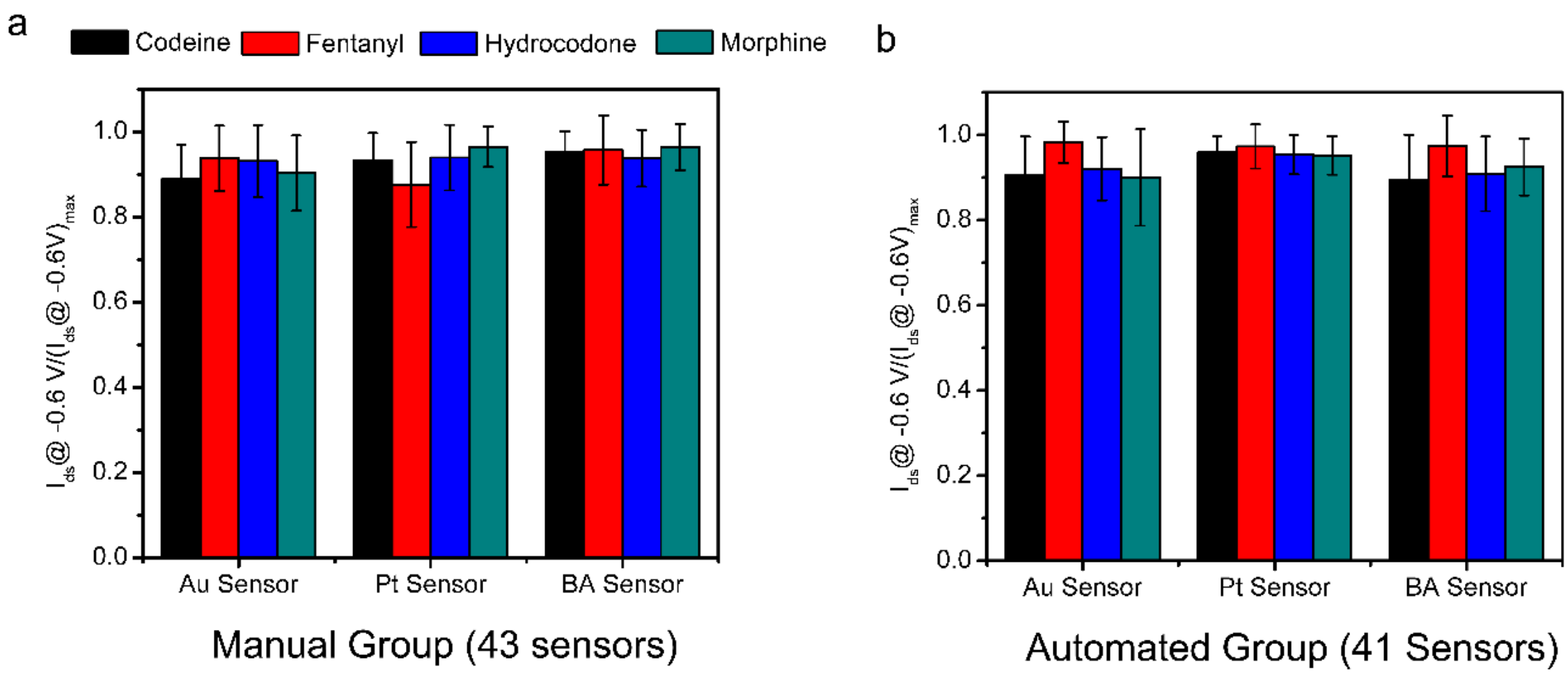

Figure S9. Stability of sensors after reuse. The evaluation is based on (a) the 43 sensors in the manual group and (b) the 41 sensors in the automated group. Source-drain current at −0.6 V before opioid incubation is used for the comparison. For each sensor, I_{ds} @−0.6 V values are recorded for each of the four opioid tests. The maximum of these four values (the maximum value in $I_{ds, Codeine}$ @−0.6 V, $I_{ds, Fentanyl}$ @−0.6 V, $I_{ds, Hydrocodone}$ @−0.6 V, and $I_{ds, morphine}$ @−0.6 V for each sensor) is designated as (I_{ds} @−0.6 V)$_{max}$ for normalization.

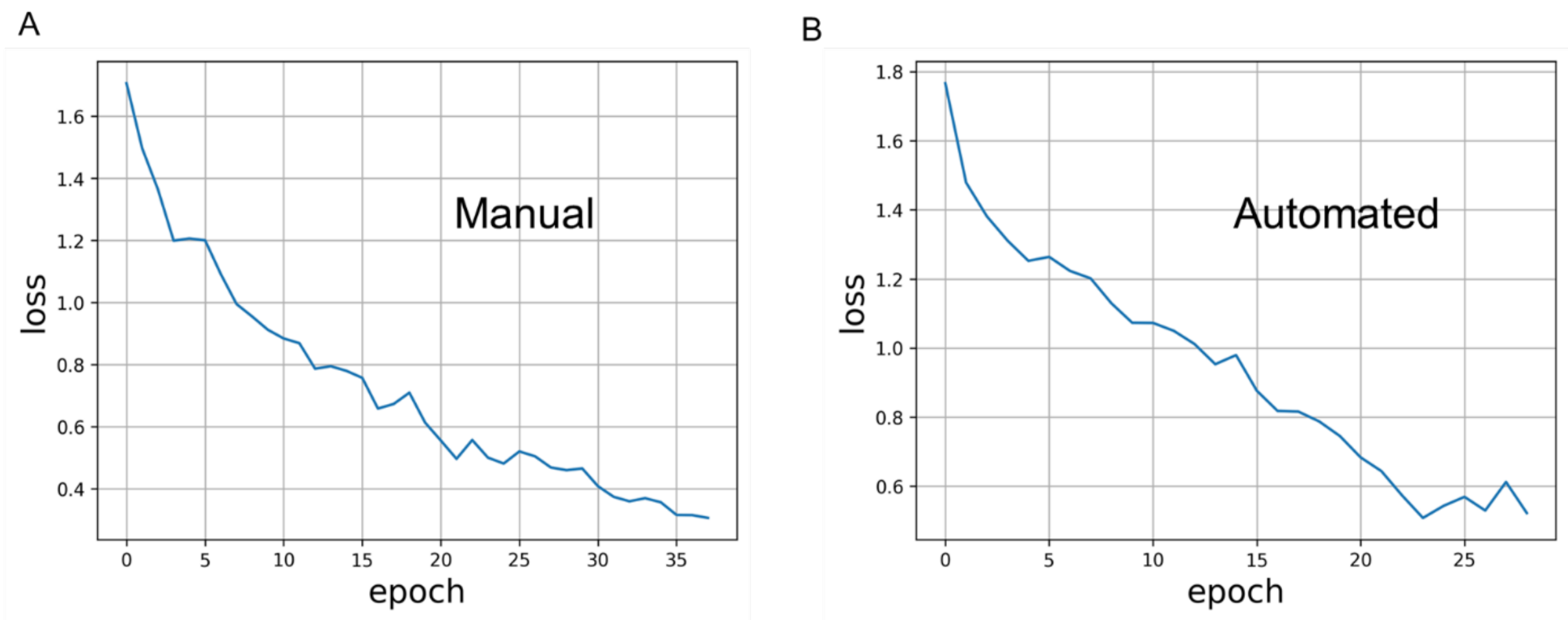

Figure S10. The training loss verse epoch curve of (A) manual and (B) automated test group using the CNN model.

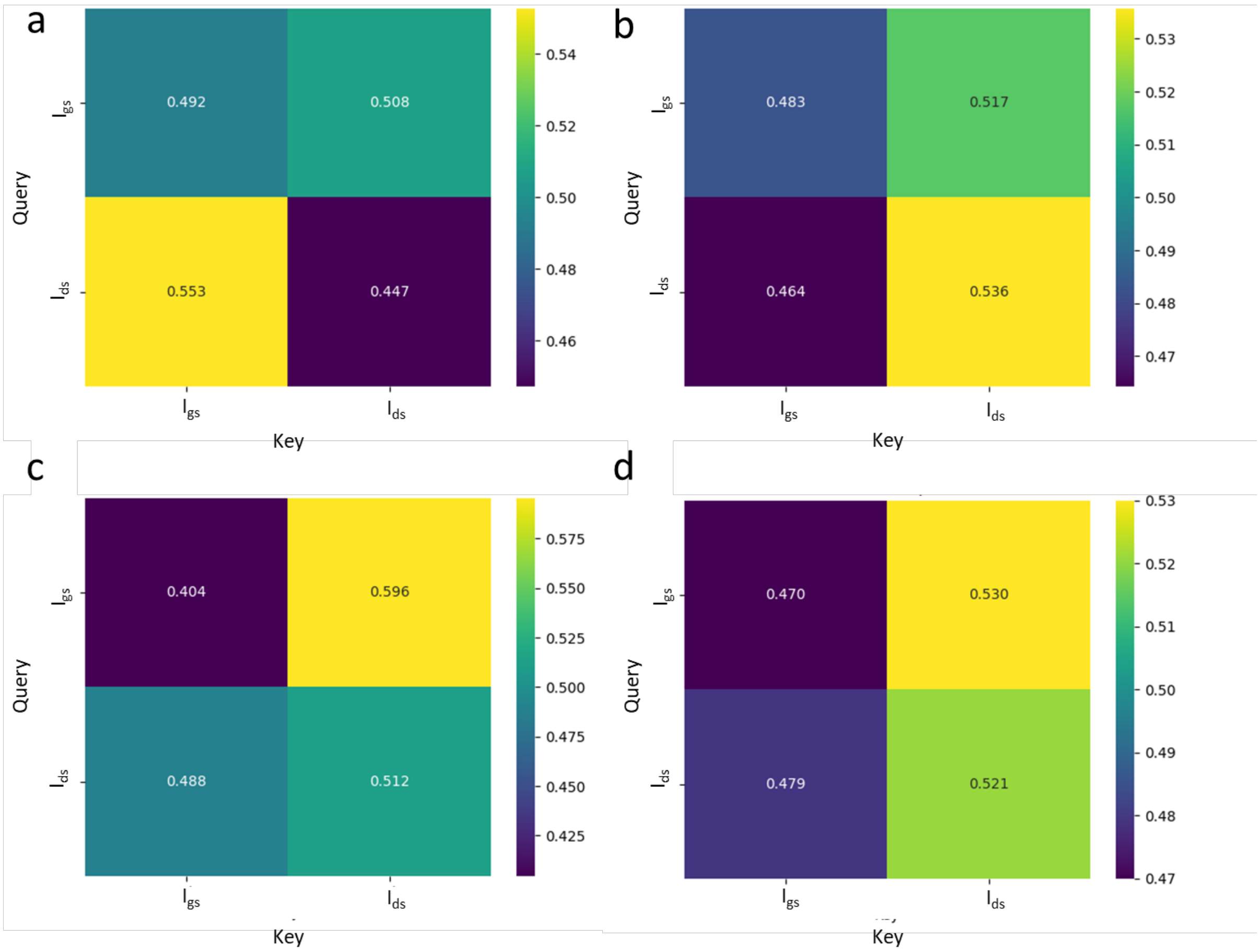

Figure S11. Attention scores of each input stream in the (a, b) manual and (c, d) automated test group on the two attention heads.

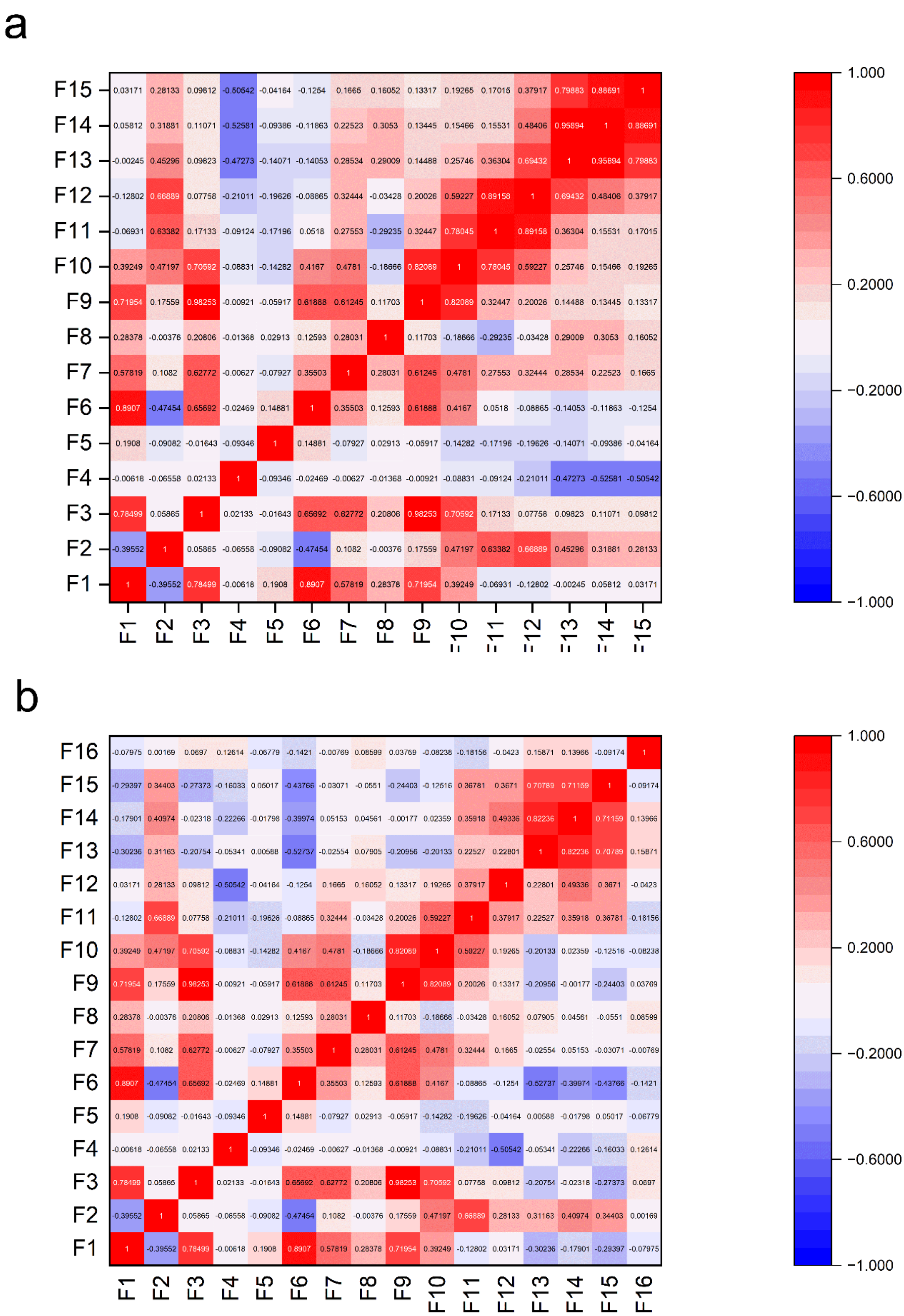

Figure S12. The Pearson correlation coefficient values between two different features in (a) the (a) original and (b) the improved conventional supervised learning model. The fentanyl sensing data collected by Au-NTFET was applied for plotting.

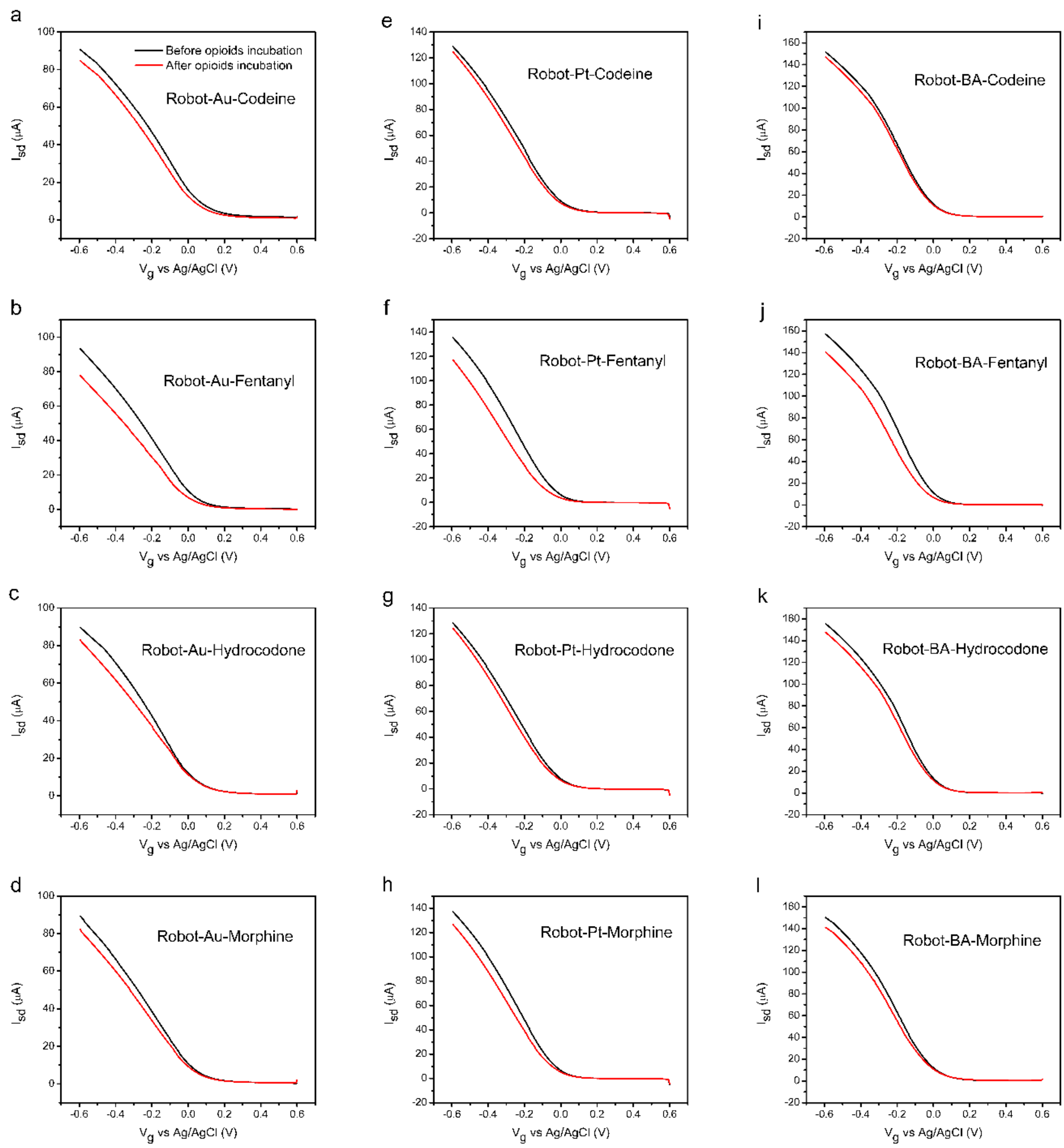

Figure S13. Transfer characteristics of NTFET, i.e, source-drain current (I_{sd}) versus applied liquid gate voltage, of differernt NTFET sensors after incubation with opioids. (a-d) Transfer characteristics of Au-NTFET after incubation with (a) codeine, (b) fentanyl, (c) hydrocodone, and (d) morphine, respectively. (e-h) Transfer characteristics of Pt-NTFET after incubation with (e) codeine, (f) fentanyl, (g) hydrocodone, and (h) morphine, respectively. (i-l) Transfer characteristics of BA-NTFET after incubation with (i) codeine, (j) fentanyl, (k) hydrocodone, and (l) morphine, respectively. Devices were incubated with phosphate buffer for 1 h as the control and opioids for another 1 h before the measurement of transfer characteristics. Drain current was measured by sweeping gate voltage (V_g) from 0.6 V to −0.6 V. Source–drain bias

voltage (V_{sd}) was 50 mV. All the data was collected with the automated electrolyte-gated FET test system.

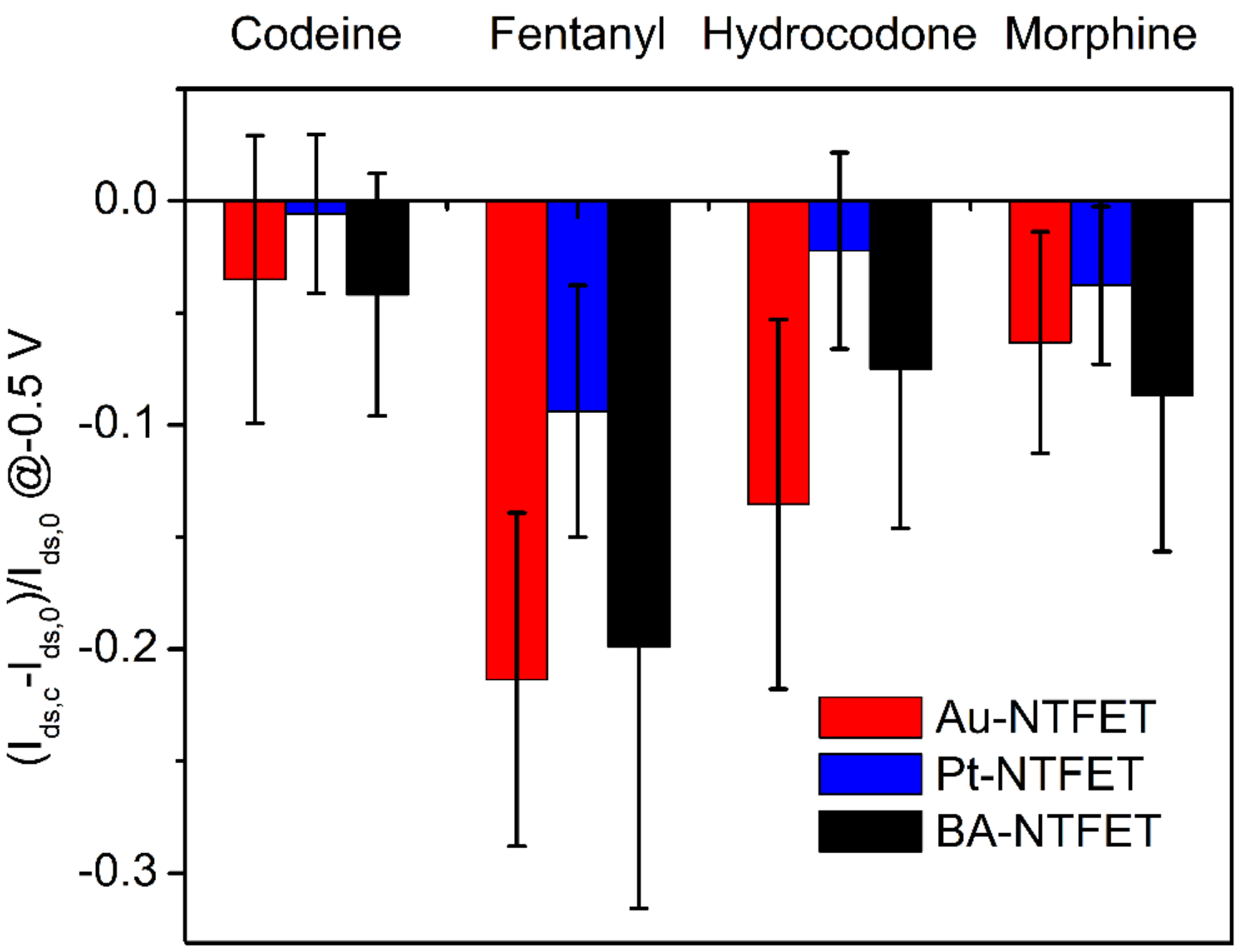

Figure S14. The changes of source-drain current at −0.5 V after incubation with different opioids on each type of sensor. All the data was collected with the automated electrolyte-gated FET test system.

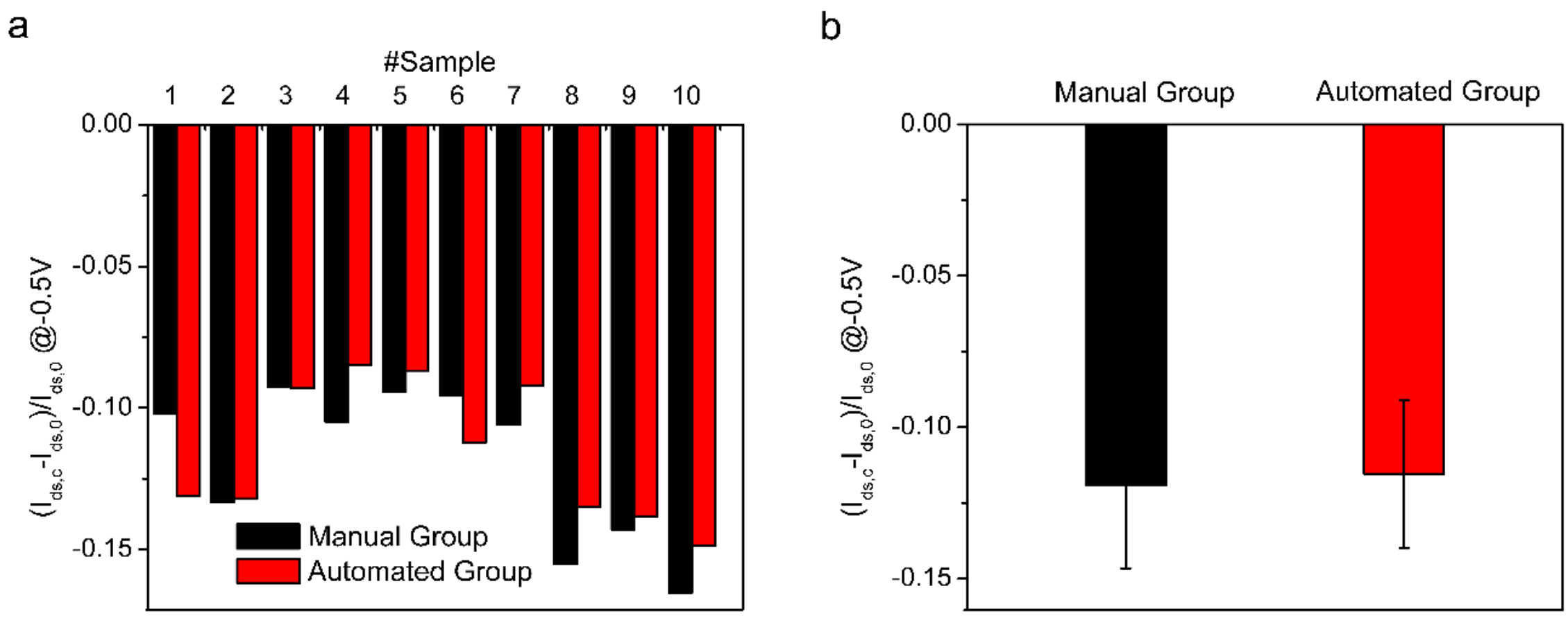

Figure S15. Changes in source-drain current at −0.5 V after incubation with fentanyl using (a) 10 different Au-NTFET sensors and (b) statistical results. Each sensor was first tested using the automated system with an additional manual drying step, then tested again manually on the following day.

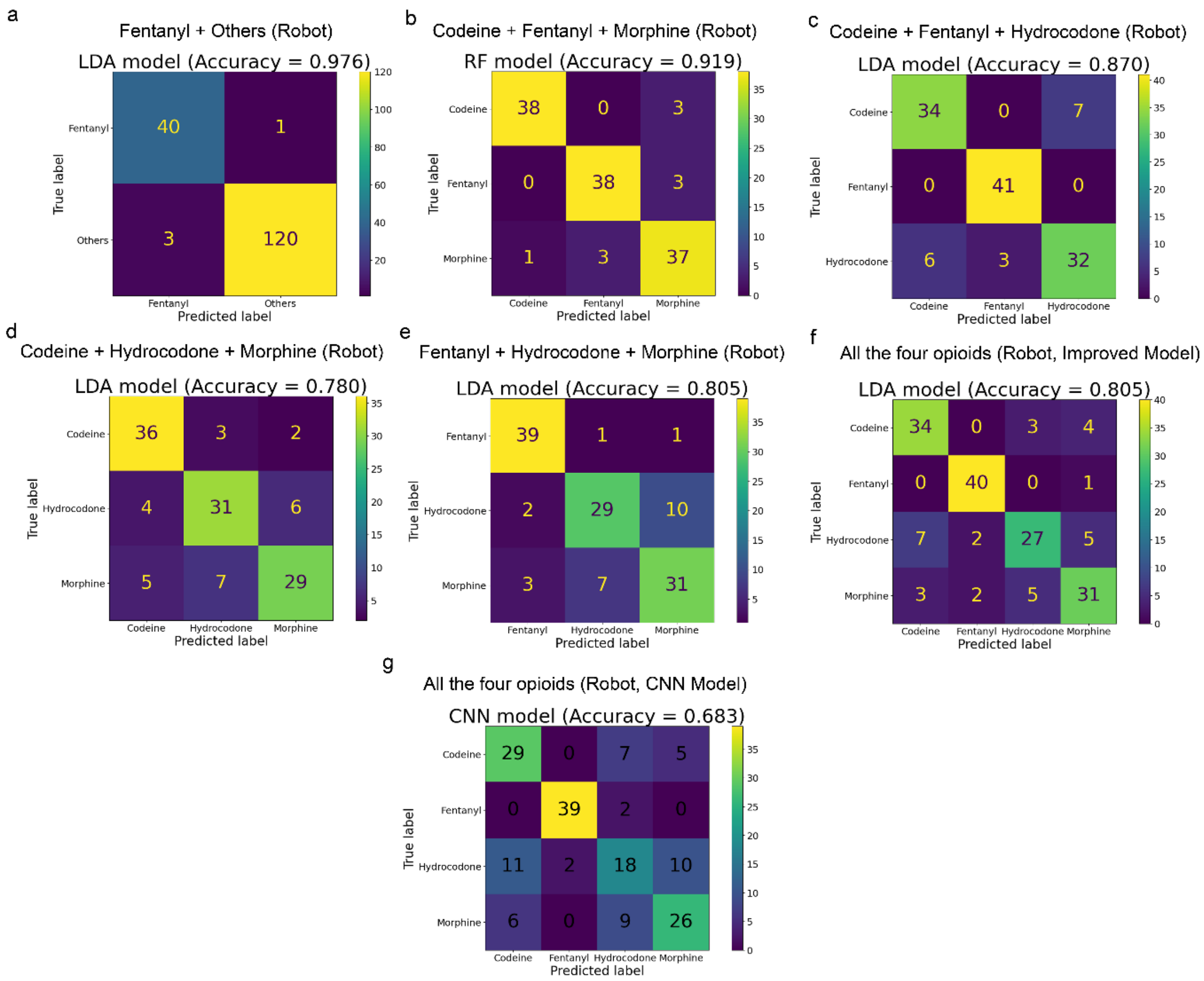

Figure S16. Confusion matrices for the automated test group, classified using conventional supervised learning models and the CNN model. (a-f) Confusion matrices for different classification tasks: (a) fentanyl vs. other opioids, (b) codeine, fentanyl and morphine, (c) codeine, fentanyl and hydrocodone, (d) codeine, hydrocodone, and morphine, (e) fentanyl, hydrocodone, and morphine, and (f) codeine, fentanyl, hydrocodone, and morphine, using the improved conventional model with features extracted from both I_{ds} and I_{gs}. (g) Confusion matrix for classification of all four opioids using the CNN model in the automated test group.

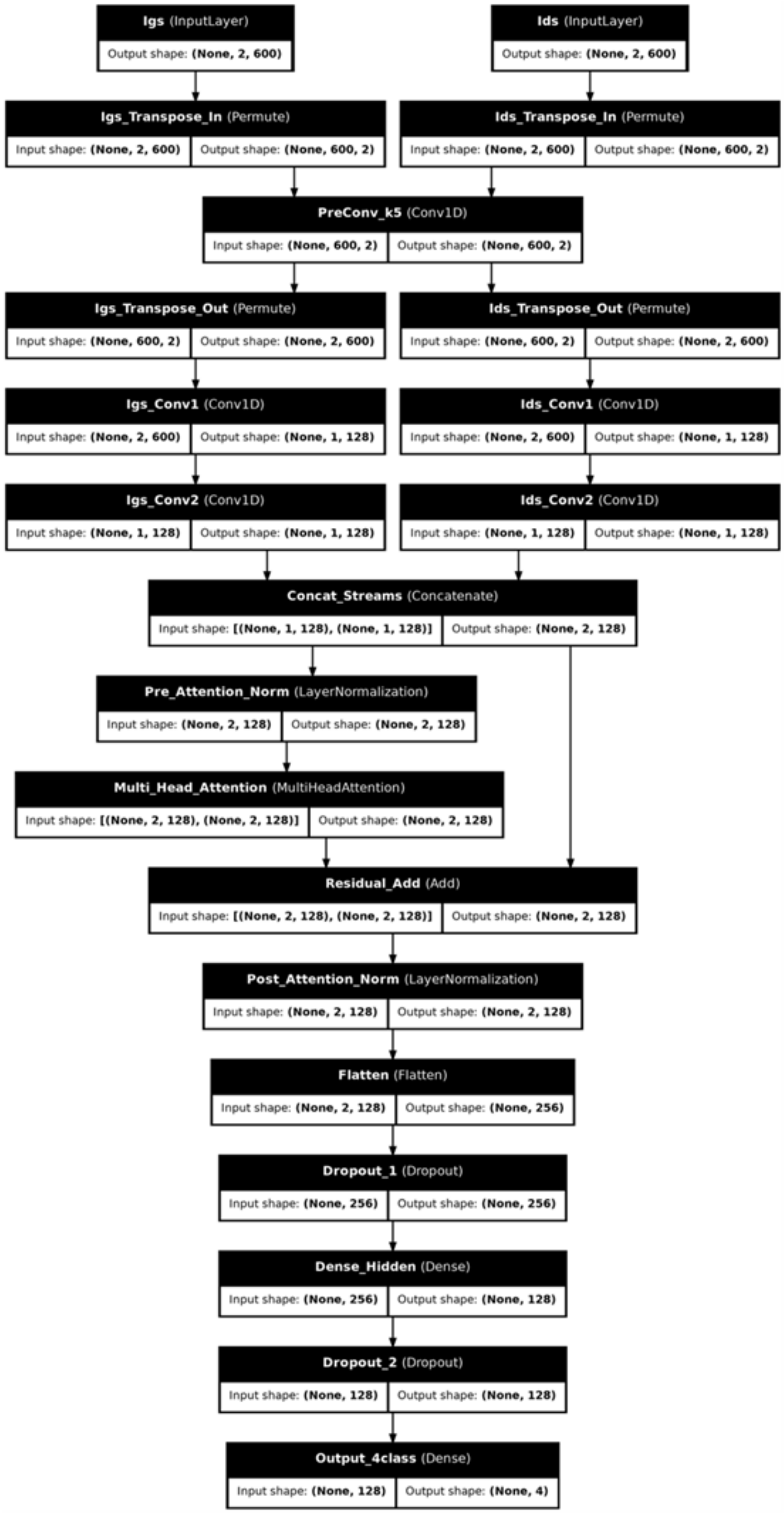

Figure S17. Architecture of the additional CNN Model A. In this model, the receptive field is increased to incorporate more global information during training.

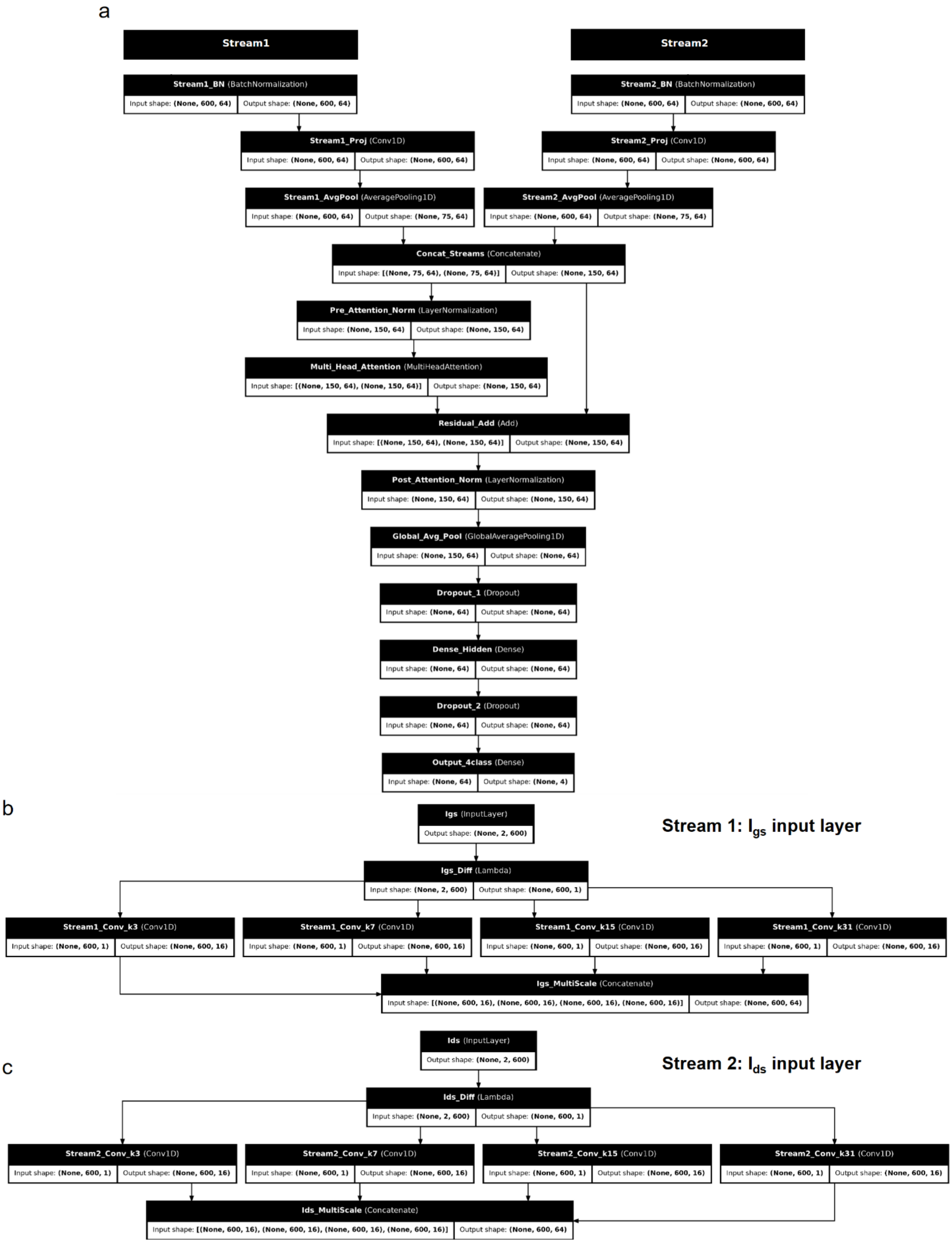

Figure S18. (a) Architecture of the additional CNN Model B, with the details of (b) Stream 1 and (c) Stream 2. In this model, superposition is assumed to occur in the raw signal domain rather than in the latent space.

Table S1. The different features in the original and improved conventional supervised learning models.

NO.	Original (I_{ds} only)	Improved (I_{ds} and I_{gs})
1[a]	$(t_c-t_0)/t_0$	$(t_c-t_0)/t_0$
2[b]	$V_{th,c}-V_{th,0}$	$V_{th,c}-V_{th,0}$
3[c]	$(I_{ds,c,max}-I_{ds,0,max})/I_{ds,0,max}$	$(I_{ds,c,max}-I_{ds,0,max})/I_{ds,0,max}$
4[d]	$(I_{ds,c,min}-I_{ds,0,min})/I_{ds,0,min}$	$(I_{ds,c,min}-I_{ds,0,min})/I_{ds,0,min}$
5[e]	$(I_{ds,c,max}/I_{ds,c,min-p})$ /$(I_{ds,0,max}/I_{ds-0,min-p})$	$(I_{ds,c,max}/I_{ds,c,min-p})$ /$(I_{ds,0,max}/I_{ds-0,min-p})$
6[f]	$I_{ds@vth-0.1v,c}/I_{ds@vth-0.1v,0}$	$I_{ds@vth-0.1v,c}/I_{ds@vth-0.1v,0}$
7[g]	$(I_{ds,c}-I_{ds,0})/I_{ds@vth}$ (-0.6 V)	$(I_{ds,c}-I_{ds,0})/I_{ds@vth}$ (-0.6 V)
8	$(I_{ds,c}-I_{ds,0})/I_{ds@vth}$ (0.3 V)	$(I_{ds,c}-I_{ds,0})/I_{ds@vth}$ (0.3 V)
9[h]	$(I_{ds,c}-I_{ds,0})/I_{ds,0}$ (–0.5 V)	$(I_{ds,c}-I_{ds,0})/I_{ds,0}$ (–0.5 V)
10	$(I_{ds,c}-I_{ds,0})/I_{ds,0}$ (–0.3 V)	$(I_{ds,c}-I_{ds,0})/I_{ds,0}$ (–0.3 V) (manual)<br>$(I_{ds,c}-I_{ds,0})/I_{ds,0}$ (–0.1 V) (Automated)
11	$(I_{ds,c}-I_{ds,0})/I_{ds,0}$ (–0.1 V)	$(I_{ds,c}-I_{ds,0})/I_{ds,0}$ (0 V)
12	$(I_{ds,c}-I_{ds,0})/I_{ds,0}$ (0 V)	$(I_{ds,c}-I_{ds,0})/I_{ds,0}$ (0.3 V)
13	$(I_{ds,c}-I_{ds,0})/I_{ds,0}$ (0.1V)	$(I_{gs,c}-I_{gs,0})/I_{gs,0}$ (–0.5 V)
14	$(I_{ds,c}-I_{ds,0})/I_{ds,0}$ (0.2 V)	$(I_{gs,c}-I_{gs,0})/I_{gs,0}$ (–0.3 V)
15	$(I_{ds,c}-I_{ds,0})/I_{ds,0}$ (0.3 V)	$(I_{gs,c}-I_{gs,0})/I_{gs,0}$ (0 V)
16[i]		$(I_{gs,c}-I_{gs,0})/I_{gs,0}$ (0.3 V)

a. The change of transconductance after opioid incubation. t is the transconductance of the sensor.

b. The shift of threshold voltage after opioid incubation.

c. The change of maximum source-drain current (I_{ds}).

d. The change of minimum I_{ds}.

e. The change of on-off ratio. The on-off ratio was estimated by the maximum I_{ds} and minimum positive I_{ds}. I_{min-p} is the minimum positive I_{ds}.

f. The change of carrier mobility. $I_{@vth-0.1v}$ is the I_{ds} at the voltage which is 0.1 V lower than the threshold voltage (in the linear region). According to the equation,

$$I_{DS} = -\frac{W}{L}\mu C\left(V_{GS} - V_{th} - \frac{V_{DS}}{2}\right)V_{DS}$$

The I_{ds} in the linear region should be propositional to carrier mobility (μ). Since W, L, and V_{DS} are constant, we can use current value around the threshold voltage to estimate the mobility. If we let V_{gs}-V_{th} = −0.1 V, we can get

$$\frac{I_{@(v_{th}-0.1\ v),sample}}{I_{@(v_{th}-0.1\ v),blank}} = \frac{\mu_{sample}}{\mu_{blank}}$$

g. $I_{@vth}$ is the I_{ds} value at the threshold voltage.

h. For Feature 9 to 15 in model 1 and Feature 9 to 12 in model 2, ($I_{ds,c}$-$I_{ds,0}$)/$I_{ds,0}$ (…V) is the change of I_{ds} at the given voltage

i. For Feature 13 to 16 in model 2, ($I_{gs,c}$-$I_{gs,0}$)/$I_{gs,0}$ (…V) is the change of gate-drain current (I_{gs}) at the given voltage

Table S2. Height profiles labeled in Figure S5a for the calculation of the diameter of bare SWCNT.

Profile	z_{max} (nm)	z_{min} (nm)	Height (nm)
1	-5.811	-6.712	0.901
2	-3.198	-4.017	0.820
3	-1.828	-3.492	1.664
4	-2.415	-3.521	1.105
5	-5.492	-6.958	1.466
6	-5.203	-7.002	1.800
7	-3.789	-5.574	1.785
8	-2.925	-4.573	1.649
9	-2.148	-2.938	0.790
10	-1.481	-2.458	0.977
11	-2.158	-2.867	0.709
12	-1.427	-2.357	0.930
13	-1.696	-2.529	0.833
14	-1.672	-2.718	1.046
15	-1.133	-2.693	1.560
16	-1.883	-3.185	1.302
17	-1.171	-3.824	2.653
18	-2.372	-4.660	2.289
19	-2.490	-4.148	1.657
20	-1.968	-3.260	1.293

Table S3. Height profiles labeled in Figure S5b for the calculation of the diameter of gold nanoparticles.

Profile	z_{max} (nm)	z_{min} (nm)	Height (nm)
1	48.917	-8.514	57.431
2	47.223	-2.553	49.776
3	54.780	-10.757	65.537
4	56.456	-0.287	56.743
5	32.442	-7.469	39.911
6	67.400	-1.981	69.380
7	41.704	1.343	40.361
8	48.166	-6.552	54.718
9	68.358	-7.718	76.077
10	58.973	-2.115	61.087
11	60.150	-3.038	63.189
12	16.908	-2.673	19.581
13	74.143	4.448	69.695
14	35.320	-2.789	38.108
15	56.844	13.208	43.635
16	67.675	7.279	60.396
17	41.340	9.383	31.958
18	52.120	-7.601	59.721
19	41.468	-11.750	53.218
20	59.316	2.605	56.711
21	61.189	-7.630	68.819
22	65.085	-3.180	68.265
23	38.582	-1.678	40.259
24	67.756	-2.767	70.523
25	21.603	-1.414	23.017
26	54.964	-6.008	60.972
27	36.265	-1.390	37.655
28	44.916	-1.330	46.246
29	57.685	-4.674	62.358
30	47.626	-2.207	49.832
31	47.405	-7.893	55.297
32	59.094	-7.635	66.729
33	43.715	-11.812	55.527
34	53.599	-12.952	66.551

Table S4. Height profiles labeled in Figure S5c for the calculation of the diameter of platinum nanoparticles.

Profile	z_{max} (nm)	z_{min} (nm)	Height (nm)
1	34.428	-0.283	34.711
2	24.960	4.119	20.841
3	30.410	8.516	21.894
4	45.300	3.585	41.715
5	73.383	19.583	53.800
6	46.969	-3.998	50.967

Table S5. Classification of four opioids in both the manual group and the automated group using different algorithms and all available features.

Algorithms	Manual Group (Original model)	Manual Group (Improved model)	Automated Group (Original model)	Automated Group (Improved model)
LDA	0.756	0.791	0.659	0.732
Linear SVM	0.587	0.692	0.512	0.555
kNN (k=5)	0.465	0.517	0.384	0.421
RF	0.750	0.860	0.671	0.695
LR	0.581	0.651	0.543	0.616

Table S6. Classification of four opioids in the manual test group based on different combinations of sensors using the original conventional supervise learning model.

Algorithm	Au	Pt	BA	Au+Pt+BA	Au+Pt+BA (After optimization)
LDA	0.616	0.610	0.587	0.756	0.826
RF	0.651	0.512	0.622	0.750	0.791

Table S7. Hyperparameter grid search results for the CNN model.

Hyperparameter	Values explored	Selected value	
		Manual Group	Automated Group
Number of filters in 1st convolution layer	64, 128, 256, 512	128	128
Number of filters in the 2nd convolution layer	64, 128, 256, 512	128	64
Number of neurons in the 1st fully connected layer	64, 128, 256, 512	128	128
Number of neurons in the 2nd fully connected layer	4	4	4
Number of heads in attention layer	1, 2	2	2
Learning rate	10^{-5}, 10^{-4}, 10^{-3}, 10^{-2}	10^{-4}	5×10^{-3}
L2 regularization parameter	10^{-7}, 10^{-6}, 10^{-5} ,10^{-4}, 10^{-3}	10^{-6}	10^{-4}

Table S8. The twenty most important I_{ds} and I_{gs} features categorized by sensor types for the classification of different opioids in the manual test group. The numbers were calculated based on the average results from each sample.

	**Au**	**Pt**	**BA**
		**I_{ds}**	
Codeine	7.22 ± 7.11	6.54 ± 6.95	6.24 ± 7.55
Fentanyl	7.28 ± 6.57	5.14 ± 6.02	7.58 ± 7.09
Hydrocodone	7.30 ± 7,14	6.21 ± 7.00	6.49 ± 6.76
Morphine	7.23 ± 6.98	5.67 ± 6.87	7.09 ± 7.04
		**I_{gs}**	
Codeine	7.78 ± 6.43	4.76 ± 6.19	7.46 ± 6.51
Fentanyl	7.63 ± 7.13	2.51 ± 4.49	9.86 ± 7.06
Hydrocodone	9.28 ± 6.51	4.77 ± 5.93	5.95 ± 6.19
Morphine	4.72 ± 5.86	5.33 ± 7.03	9.95 ± 7.72

Table S9. Features remained in the improved coventional supervised learning models after elimination with RFECV method. All the data was manually collected. The leave-one-out method was applied for cross-validation.

Classification	Selected features		1	2	3	4	5	6	7	8	9	10	11	12	13	14	15	16	Accuracy
Fentanyl and others	33	Au	✓	✓	✓			✓		✓	✓	✓	✓		✓	✓		✓	0.988 (LDA)
		Pt	✓	✓	✓	✓		✓	✓	✓	✓	✓	✓					✓	
		BA	✓	✓	✓			✓	✓	✓	✓	✓			✓	✓		✓	
Codeine, Fentanyl, and Morphine	20	Au		✓	✓				✓		✓	✓	✓		✓	✓		✓	0.961 (RF)
		Pt		✓	✓				✓		✓	✓						✓	
		BA		✓									✓		✓	✓		✓	
Codeine, Fentanyl, and Hydrocodone	26	Au		✓	✓						✓	✓	✓		✓	✓	✓	✓	0.953 (RF)
		Pt	✓		✓				✓		✓	✓	✓		✓	✓		✓	
		BA	✓	✓				✓		✓		✓	✓		✓			✓	
Codeine, Hydrocodone, and Morphine	48	Au	✓	✓	✓	✓	✓	✓	✓	✓	✓	✓	✓	✓	✓	✓	✓	✓	0.907 (RF)
		Pt	✓	✓	✓	✓	✓	✓	✓	✓	✓	✓	✓	✓	✓	✓	✓	✓	
		BA	✓	✓	✓	✓	✓	✓	✓	✓	✓	✓	✓	✓	✓	✓	✓	✓	
Fentanyl, Hydrocodone, and Morphine	41	Au	✓	✓	✓	✓			✓		✓	✓	✓	✓		✓	✓	✓	0.875 (RF)
		Pt	✓	✓	✓	✓		✓	✓	✓	✓	✓	✓	✓	✓	✓	✓	✓	
		BA	✓	✓	✓	✓	✓	✓	✓		✓	✓	✓	✓	✓	✓		✓	
Codeine, Fentanyl, Hydrocodone, and Morphine	32	Au	✓	✓	✓				✓		✓	✓	✓		✓	✓	✓	✓	0.890 (RF)
		Pt	✓	✓	✓				✓	✓	✓	✓	✓		✓	✓		✓	
		BA	✓	✓	✓	✓						✓	✓		✓	✓	✓	✓	

Table S10. The twenty most important I_{ds} and I_{gs} features categorized by sensor types for the classification of different opioids in the automated test group. The numbers were calculated based on the average results from each sample.

	Au	Pt	BA
		**I_{ds}**	
Codeine	4.41 ± 5.39	9.66 ± 6.42	5.93 ± 6.32
Fentanyl	5.63 ± 6.41	6.90 ± 6.22	7.46 ± 6.98
Hydrocodone	4.51 ± 6.08	8.07 ± 6.18	7.41 ± 6.65
Morphine	5.68 ± 6.43	7.95 ± 7.40	6.38 ± 7.58
		**I_{gs}**	
Codeine	7.37 ± 6.14	5.46 ± 6.47	7.17 ± 6.70
Fentanyl	6.61 ± 5.73	4.76 ± 5.57	8.63 ± 6.76
Hydrocodone	5.95 ± 5.70	6.39 ± 6.57	7.66 ± 6.46
Morphine	7.78 ± 6.41	6.15 ± 6.47	6.08 ± 5.39

Table S11. Features remained in the improved coventional supervised learning models after elimination with RFECV method. All the data was collected with the automated system. The leave-one-out method was applied for cross-validation.

Classification	Selected features		1	2	3	4	5	6	7	8	9	10	11	12	13	14	15	16	Accuracy
		Au	✓	✓				✓		✓	✓		✓					✓	
Fentanyl and others	26	Pt	✓	✓	✓			✓		✓	✓	✓	✓		✓			✓	0.975 (LDA)
		BA	✓	✓	✓			✓			✓	✓	✓		✓	✓			
		Au			✓				✓		✓	✓							
Codeine, Fentanyl, and Morphine	12	Pt			✓				✓		✓							✓	0.919 (RF)
		BA			✓				✓		✓							✓	
		Au	✓	✓	✓					✓	✓		✓						
Codeine, Fentanyl, and Hydrocodone	20	Pt	✓	✓	✓			✓			✓	✓	✓					✓	0.870 (LDA)
		BA	✓	✓	✓						✓	✓	✓						
		Au	✓	✓	✓			✓		✓	✓	✓	✓						
Codeine, Hydrocodone, and Morphine	27	Pt	✓	✓	✓			✓		✓	✓	✓	✓					✓	0.780 (LDA)
		BA	✓	✓	✓			✓	✓	✓	✓	✓	✓					✓	
		Au	✓	✓	✓			✓		✓	✓		✓						
Fentanyl, Hydrocodone, and Morphine	22	Pt	✓	✓	✓			✓		✓	✓	✓	✓					✓	0.805 (LDA)
		BA	✓	✓	✓						✓	✓	✓						
		Au	✓	✓	✓			✓		✓	✓	✓	✓						
Codeine, Fentanyl, Hydrocodone, and Morphine	22	Pt	✓	✓	✓					✓	✓	✓	✓					✓	0.805 (LDA)
		BA	✓	✓	✓						✓	✓	✓						

Table S12. Classification report for the original model, improved model, and CNN model for both the manual group and the automated group.

True Label \ Predicted	Codeine	Fentanyl	Hydrocodone	Morphine	Precision	Recall	F1
**Original model (Manual Group)**							
Codeine	40	0	0	3	0.833	0.930	0.879
Fentanyl	0	39	3	1	0.929	0.907	0.918
Hydrocodone	5	2	30	6	0.769	0.698	0.732
Morphine	3	1	6	33	0.767	0.767	0.767
Macro Precision = 0.825, Macro Recall = 0.826, Macro F1 =0.824							
**Improved model (Manual Group)**							
Codeine	41	1	0	1	0.932	0.953	0.943
Fentanyl	0	41	2	0	0.911	0.953	0.932
Hydrocodone	1	2	35	5	0.854	0.814	0.833
Morphine	2	1	4	36	0.857	0.837	0.847
Macro Precision = 0.888, Macro Recall = 0.890, Macro F1 =0.889							
**CNN model (Manual Group)**							
Codeine	43	0	0	0	1	1	1
Fentanyl	0	42	0	1	0.977	0.977	0.977
Hydrocodone	0	0	43	0	0.977	1	0.989
Morphine	0	1	1	41	0.976	0.953	0.965
Macro Precision = 0.983, Macro Recall = 0.983, Macro F1 =0.982							

Original model (Automated Group)

Codeine	32	0	3	6	0.681	0.780	0.727
Fentanyl	0	39	1	1	0.929	0.951	0.940
Hydrocodone	8	3	18	12	0.692	0.439	0.537
Morphine	7	0	4	30	0.612	0.732	0.667

Macro Precision = 0.728, Macro Recall = 0.726, Macro F1 =0.718

Improved model (Automated Group)

Codeine	34	0	3	4	0.773	0.829	0.8
Fentanyl	0	40	0	1	0.909	0.976	0.941
Hydrocodone	7	2	27	5	0.771	0.659	0.711
Morphine	3	2	5	31	0.756	0.756	0.756

Macro Precision = 0.802, Macro Recall = 0.805, Macro F1 =0.802

CNN (Automated Group)

Codeine	29	0	7	5	0.630	0.707	0.667
Fentanyl	0	39	2	0	0.951	0.951	0.951
Hydrocodone	11	2	18	10	0.5	0.439	0.468
Morphine	6	0	9	26	0.634	0.634	0.634

Macro Precision = 0.679, Macro Recall = 0.683, Macro F1 =0.680

Table S13. Classification results for the manual group and the automated group using the CNN model validated on the same sensor combinations as the conventional models.

Dropout rate / Learning rate	0.2	0.3	0.4
		**Validation Accuracy**	
		**Manual Group (99 epochs)**	
5×10^{-3}			0.91
		**Automated Group (>100 epochs)**	
10^{-2}	0.63	0.64	0.61
10^{-3}	0.63	0.65	0.66
10^{-6}	0.59	0.58	0.58

Appendix A. Python Codes for the Classification of Opioids with Leave-one-out Cross-Validation.

```
from sklearn.ensemble import RandomForestClassifier
from sklearn.discriminant_analysis import LinearDiscriminantAnalysis
from sklearn.model_selection import LeaveOneOut
from sklearn.metrics import confusion_matrix
from sklearn.model_selection import cross_validate
from sklearn.model_selection import cross_val_predict
from sklearn.metrics import ConfusionMatrixDisplay
import matplotlib.pyplot as plt
import pandas as pd
data = pd.read_csv("/RFECV/Robot classification.csv")
X=
data[['F1','F2','F3','F4','F5','F6','F7','F8','F9','F10','F11','F12','F13','F14','F15','F16','F17','F18','F19','F20'
,'F21','F22','F23','F24','F25','F26','F27','F28','F29','F30','F31','F32','F33','F34','F35','F36','F37','F38','F39
','F40','F41','F42','F43','F44','F45','F46','F47','F48']].values
y=data['target'].values

LDA = LinearDiscriminantAnalysis()
RF = RandomForestClassifier(random_state=0)

cv_results_LDA = cross_validate(LDA, X, y,cv=LeaveOneOut(), scoring=["accuracy"])
cv_results_RF= cross_validate(RF, X, y,cv=LeaveOneOut(), scoring=["accuracy"])

LDA_accuracy=cv_results_LDA['test_accuracy'].mean()
RF_accuracy=cv_results_RF['test_accuracy'].mean()


#LDA results
print("The LDA accuracy is: %f"%LDA_accucacy)
LDA_y_pred = cross_val_predict(LDA, X, y,cv=LeaveOneOut())
LDA_ax = plt.figure(figsize=(10, 5)).subplots()
LDA_ax.set(title="Validation Accuracy with LDA")
ConfusionMatrixDisplay.from_predictions(y, LDA_y_pred,ax=LDA_ax)

#RF results
print("The RF accuracy is: %f"%RF_accucacy)
RF_y_pred = cross_val_predict(RF, X, y,cv=LeaveOneOut())
RF_ax = plt.figure(figsize=(10, 5)).subplots()
RF_ax.set(title="Validation Accuracy with RF")
ConfusionMatrixDisplay.from_predictions(y, RF_y_pred,ax=RF_ax)
```

Appendix B. Python Codes for the Opioid Sensing with an Automated Electrolyte-gated FET Test System.

```
import json
import requests
from opentrons import protocol_api, types

metadata = {
    'protocolName': 'opioid sensing',
    'author': 'Zhengru Liu',
    'description': 'This protocol is for sensing experiments using the incubation method',
    'apiLevel': '2.14'}

def run(protocol: protocol_api.ProtocolContext):
    PBS = protocol.load_labware('generic_3_reservoir_65ml', 6)
    waste = protocol.load_labware('nest_12_reservoir_15ml', 11)
    sample = protocol.load_labware('corning_96_wellplate_360ul_flat', 3)
    tiprack_1000= protocol.load_labware('opentrons_96_tiprack_1000ul', 10)
    tiprack_300 = protocol.load_labware('opentrons_96_tiprack_300ul', 9)
    p1000 = protocol.load_instrument('p1000_single', 'right', tip_racks=[tiprack_1000])
    p300 = protocol.load_instrument('p300_single', 'left', tip_racks=[tiprack_300])
    sens_a = protocol.load_labware('new_device_well_pos7', '7')
    sens_b = protocol.load_labware('new_device_well_pos8', '8')
    sens_c = protocol.load_labware('new_device_well_pos4', '4')
    sens_d = protocol.load_labware('new_device_well_pos5', '5')
    sens_e = protocol.load_labware('new_device_well_pos1', '1')
    sens_f = protocol.load_labware('new_device_well_pos2', '2')

    sens1,sens2,sens3,sens4,sens5,sens6,sens7,sens8,sens9,sens10,sens11,sens12 =
sens_a["C3"],sens_a["C22"],sens_b["C3"],sens_b["C22"],sens_c["C3"],sens_c["C22"],sens_d["C3"],s
ens_d["C22"],sens_e["C3"],sens_e["C22"],sens_f["C3"],sens_f["C22"]


    senslist = [sens1,sens2,sens3,sens4,sens5,sens6,sens7,sens8,sens9,sens10,sens11,sens12]
    cleanlist= [sens_a,sens_b,sens_c,sens_d,sens_e,sens_f]

#rinsing step before incubation (wash for three times)
    for i in range(3):
        p300.distribute(240,PBS["A1"],senslist[0:9],disposal_volume=0)
        protocol.delay(minutes=10)
        p300.consolidate(300,senslist[0:9],waste["A1"])
        i+=1
```

```
# incubated with PBS for 1 h
    p300.distribute(240,PBS["A1"],senslist[0:9],disposal_volume=0)
    protocol.delay(minutes=60)
    p300.consolidate(300,senslist[0:9],waste["A1"])

# rinsing step
    p300.distribute(240,PBS["A1"],senslist[0:9],disposal_volume=0)
    protocol.delay(minutes=1)
    p300.consolidate(300,senslist[0:9],waste["A1"])

# FET measurement
    for i in range(9):
        # add gating liquid
            p300.transfer(240,PBS["A2"],senslist[i])
        # pick the reference electrode
            p1000.pick_up_tip(tiprack_1000['A1'].top(z=18))
        # move reference electrode to the device
            p1000.move_to(senslist[i].bottom(z=1.2))
            protocol.delay(minutes=2)
            if not protocol.is_simulating():
                done = False
                while not done:
                    r = requests.post('http:// *.*.*.*:*/update', json={'step': 'moved_to_sensor', 'work':
"OPI-BLK", 'position': i, 'channel': int(1 + i * 8)})
                    done = r.json()['done']
        # please type in the correct address of the host
        # return the reference electrode
            p1000.return_tip()
            p300.transfer(300,senslist[i],waste["A1"])

# solution removal
    p300.pick_up_tip()
    for i in range (5):
        for j in range(5):
            for k in range(3):
                p300.aspirate(20,cleanlist[i].columns()[j][k].bottom(z=0.3))
        p300.dispense(300,waste["A1"])
        for j in range(5):
            for k in range(3,5):
                p300.aspirate(20,cleanlist[i].columns()[j][k].bottom(z=0.3))
        p300.dispense(300,waste["A1"])

        for m in range(5):
            for k in range(3):
```

```
            p300.aspirate(20,cleanlist[i].columns()[m+19][k].bottom(z=0.3))
        p300.dispense(300,waste["A1"])
        for m in range(5):
            for k in range(3,5):
                p300.aspirate(20,cleanlist[i].columns()[m+19][k].bottom(z=0.3))
        p300.dispense(300,waste["A1"])

    p300.drop_tip()



# incubation with opioids solution
# (sample["A1"]="codeine", sample["A2"]="hydrocodone", sample["A3"]="morphine")
    p300.distribute(100,sample["A1"].bottom(z=0.5),senslist[0:3],disposal_volume=0)
    p300.distribute(100,sample["A2"].bottom(z=0.5),senslist[3:6],disposal_volume=0)
    p300.distribute(100,sample["A3"].bottom(z=0.5),senslist[6:9],disposal_volume=0)
    protocol.delay(minutes=60)

# remove opioids solution
    p300.consolidate(200,senslist[0:3],sample["C1"],blow_out="True")
    p300.consolidate(200,senslist[3:6],sample["C2"],blow_out="True")
    p300.consolidate(200,senslist[6:9],sample["C3"],blow_out="True")
    for i in range(5):
        p300.pick_up_tip()
        for j in range(5):
            for k in range(3):
                p300.aspirate(20,cleanlist[i].columns()[j][k].bottom(z=0.3))
        p300.dispense(300,waste["A1"])
        for j in range(5):
            for k in range(3,5):
                p300.aspirate(20,cleanlist[i].columns()[j][k].bottom(z=0.3))
        p300.dispense(300,waste["A1"])
        p300.drop_tip()

        p300.pick_up_tip()
        for m in range(5):
            for k in range(3):
                p300.aspirate(20,cleanlist[i].columns()[m+19][k].bottom(z=0.3))
        p300.dispense(300,waste["A1"])
        for m in range(5):
            for k in range(3,5):
                p300.aspirate(20,cleanlist[i].columns()[m+19][k].bottom(z=0.3))
        p300.dispense(300,waste["A1"])
        p300.drop_tip()
```

```
# rinsing step
    for i in range(2):
        p300.distribute(240,PBS["A1"],senslist[0:9],disposal_volume=0)
        protocol.delay(minutes=1)
        p300.consolidate(300,senslist[0:9],waste["A1"])
        i+=1

# FET measurement
    for i in range(9):
        # add gating liquid
            p300.transfer(240,PBS["A2"],senslist[i])
        # pick the reference electrode
            p1000.pick_up_tip(tiprack_1000['A1'].top(z=18))
        # move reference electrode to the device
            p1000.move_to(senslist[i].bottom(z=1.2))
            protocol.delay(minutes=2)
            if not protocol.is_simulating():
                done = False
                while not done:
                    r = requests.post('http:// *.*.*.*:*/update', json={'step': 'moved_to_sensor', 'work':
"OPI", 'position': i, 'channel': int(1 + i * 8)})
                    done = r.json()['done']
        # please type in the correct address of the host
        # return the reference electrode
            p1000.return_tip()
            p300.transfer(300,senslist[i],waste["A1"])
```
